# Ubiquitin-dependent degradation of p27^Kip1^ and p21^Waf1/Cip1^ by AMBRA1 ensures G1 and S phase progression and limits replication stress

**DOI:** 10.1093/nar/gkag595

**Published:** 2026-06-24

**Authors:** Giacomo Milletti, Giulia Cadeddu, Alba Adelantado-Rubio, Caterina Ferraina, Kristina Keuper, Sebastian Howen Nesgaard Munk, Cristiano De Stefanis, Denise Quacquarini, Sabrina Rossi, Angela Mastronuzzi, Franco Locatelli, Paolo Grumati, Valentina Cianfanelli, Francesca Nazio, Jiri Bartek, Apolinar Maya-Mendoza, Francesco Cecconi

**Affiliations:** DNA Replication and Cancer Group, Danish Cancer Institute, 2100Copenhagen, Denmark; Dipartimento di Scienze biotecnologiche di base, cliniche intensivologiche e perioperatorie, Università Cattolica del Sacro Cuore, 00168Rome, Italy; Fondazione Policlinico Universitario Agostino Gemelli IRCCS, 00168Rome, Italy; DNA Replication and Cancer Group, Danish Cancer Institute, 2100Copenhagen, Denmark; Dipartimento di Scienze biotecnologiche di base, cliniche intensivologiche e perioperatorie, Università Cattolica del Sacro Cuore, 00168Rome, Italy; DNA Replication and Cancer Group, Danish Cancer Institute, 2100Copenhagen, Denmark; DNA Replication and Cancer Group, Danish Cancer Institute, 2100Copenhagen, Denmark; Research Facilities, Bambino Gesù Children’s Hospital IRCCS, 00165Rome, Italy; Pathology Unit, Bambino Gesù Children’s Hospital IRCCS, 00165Rome, Italy; Pathology Unit, Bambino Gesù Children’s Hospital IRCCS, 00165Rome, Italy; Hematology/Oncology, Cell Therapy, Gene Therapies and Hemopoietic Transplant, Bambino Gesù Children’s Hospital IRCCS, 00165Rome, Italy; Department of Health Science and Public Health, Catholic University of the Sacred Heart, 00168Rome, Italy; Hematology/Oncology, Cell Therapy, Gene Therapies and Hemopoietic Transplant, Bambino Gesù Children’s Hospital IRCCS, 00165Rome, Italy; Department of Health Science and Public Health, Catholic University of the Sacred Heart, 00168Rome, Italy; Telethon Institute of Genetics and Medicine, Pozzuoli, 80078Naples, Italy; Department of Science, University “ROMA TRE”, 00146 Rome, Italy; Department of Biology, University of Rome Tor Vergata, 00133 Rome, Italy; Genome Integrity Group, Danish Cancer Institute, 2100Copenhagen, Denmark; Division of Genome Biology, Department of Medical Biochemistry and Biophysics, Science for Life Laboratory, Karolinska Institutet, S-171 21 Stockholm, Sweden; Division of Cancer Research, Ninewells Hospital and Medical School, University of Dundee, Dundee DD19SY, United Kingdom; DNA Replication and Cancer Group, Danish Cancer Institute, 2100Copenhagen, Denmark; Dipartimento di Scienze biotecnologiche di base, cliniche intensivologiche e perioperatorie, Università Cattolica del Sacro Cuore, 00168Rome, Italy; Gemelli Science and Technology Park (GSTeP) - BI-Org Research Core Facility, Fondazione Policlinico Agostino Gemelli IRCCS, 00168 Rome, Italy

## Abstract

The cell cycle orchestrates the events that lead to cell replication and division. AMBRA1 interacts with the E3 ubiquitin ligase CRL4^DDB1^ complex to regulate the stability of D-type cyclins, key regulators of the G1–S phase transition. However, whether AMBRA1 has a role in the S phase remains to be elucidated. Here, we show that AMBRA1 affects the turnover of p21^Waf1/Cip1^ and p27^Kip1^ by coupling the CRL4^DDB1^ complex to these proteins. In the absence of AMBRA1, the increased stability of p21^Waf1/Cip1^, rather than p27^Kip1^, resulted in the accumulation of replication stress. Mechanistically, the excess of p21^Waf1/Cip1^ during the S phase resulted in more PCNA-bound p21^Waf1/Cip1^, negatively affecting the binding of FEN1 to PCNA, which left under-replicated DNA. Consequently, AMBRA1-depleted cells are sensitive to FEN1 inhibition. Aberrantly low levels of AMBRA1 occurring in Sonic Hedgehog-type medulloblastomas correlate with high levels of p21^Waf1/Cip1^ and a worse prognosis. Finally, AMBRA1 and p21 levels may serve as biomarkers for patient stratification and treatment in medulloblastoma.

## Graphical Abstract

**Graphical Abstract:**
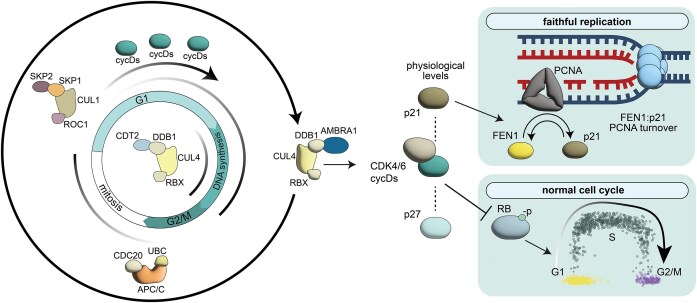

## Introduction

The orchestration of the cell cycle is determined by growth factors and metabolic signals, which are ultimately integrated by cyclins (including cyclins A, B, C, D, and E) and cyclin-dependent kinases (CDKs). Specific cyclins regulate transitions through the cell cycle through processes of synthesis and degradation. D-type cyclins in complex with CDK4/6 drive the transition from G1 to S phase. The accumulation of cyclin D1 due to aberrant regulation results in a fast cell cycle, increased speed of DNA replication fork progression, and genome instability [1]. Several other proteins and post-translational modifications act in concert to regulate each phase of the cell cycle; classical examples of those are p21^Waf1/Cip1^ and p27^Kip1^ (hereafter referred as p21 and p27) [2–5]. Among several functions, p27 and p21 inhibit the activity of CDK2-containing complexes and serve as assembly factors for cyclin D–CDK4/6 [6, 7]. The latter role was demonstrated by the observation that mouse fibroblasts lacking p27 and p21 fail to assemble cyclin D–CDK4/6 complexes [8].

The Activating Molecule in Beclin 1-Regulated Autophagy (AMBRA1) acts as a substrate receptor for CRL4^DDB1^-mediated degradation of D-type cyclins, restraining G1/S transition and thereby preserving genome integrity [1, 9]. Cullin-RING finger ligases (CRLs) are the largest family of ubiquitin ligases in eukaryotic cells, regulating diverse cellular processes. Unlike other cullins, CUL4 (CUL4A and CUL4B) employs DDB1, a WD40-like repeat-containing adaptor, to assemble the CRL4^DDB1^ complex [10]. This E3 ubiquitin ligase governs protein turnover through ubiquitination, playing a crucial role in DNA replication, repair, and cell cycle control, thereby ensuring cellular homeostasis [11, 12]. Numerous E3 ubiquitin ligases have been implicated in the recognition of p27 and p21; however, the full regulatory network governing their turnover remains to be fully elucidated. Understanding these mechanisms is essential for deciphering cell cycle control and its impact on genomic stability.

Growing evidence highlights the crucial role of AMBRA1 in cancer development. Notably, AMBRA1 expression varies among different medulloblastoma (MB) subgroups [13], with the lowest levels observed in Sonic Hedgehog–MB (MB-SHH), a subtype characterized by high genomic instability [14]. This instability is associated with a replication stress (RS) phenotype similar to that caused by AMBRA1 depletion [15]. Moreover, a Sleeping Beauty (SB) transposon mutagenesis analysis identified AMBRA1 as a novel genetic driver of embryonal brain tumors primed by p53 loss [16].

Here, we show that AMBRA1 coordinates the degradation of p27 and p21, besides that of D-type cyclins. Indeed, AMBRA1 modulates genomic DNA synthesis indirectly by maintaining adequate levels of p21. Loss of AMBRA1 leads to p21 accumulation, which induces RS by misregulated replication fork progression. Mechanistically, excess p21 binds to PCNA, displacing the PCNA-interacting endonuclease FEN1, an enzyme essential for Okazaki fragment processing during lagging-strand synthesis. This displacement impairs lagging-strand maturation, resulting in under-replicated DNA and genomic instability. Further, we here elucidate the role of AMBRA1 as a tumor suppressor in p53-null MB-SHH cell lines, where the permissive environment generated by p53 mutation enables AMBRA1-depleted cells to cope with elevated RS.

Lastly, our data shed light on the therapeutically relevant role of AMBRA1 in modulating the response to CDK4/6 inhibitors as well as inhibiting the lagging-strand DNA synthesis in MB-SHH cell lines [17, 18], thus heralding potential innovative treatment avenues for this lethal disease.

## Materials and methods

### Generation of Ambra1^flox/flox^/Nestin-cre mice

To generate Ambra1^flox/flox^/Nestin-cre [1], homozygous Ambra1^flox/flox^ females were bred onto Ambra1^wt/flox^/Nestin-cre. Nestin-cre were purchased from Jackson Lab and maintained in the animal facility Plaisant Castel Romano (RM, Italy). Mutants were genotyped by polymerase chain reaction (PCR) of genomic DNA extracted from tails or ears using the following primers:

*Tm1c* FW 5′-TGATAGTCCACGCTCGACCT-3′,
*Tm1c* RV 5′-CTAATCCGCCTACTGCGACT-3′,
*Ambra1* wt FW 5′-TCTGGTTGCCTAGATGGGGA-3′,
A*mbra1* wt RV 5′-ACTCATGTTAGAGCCTCCTGC-3′,
*Cre* FW 5′-CGGTCGATGCAACGAGTGATGAGG-3′,
*Cre* RV 5′-CCAGAGACGGAAATCCATCGCTCG-3′,

All *in vivo* experiments were approved by and performed following the ethical international, EU, and national requirements and were approved by the Italian Health Ministry (D.lgs 26/2014; N°. 737 03/2013; N°88/2016-PR).

### Cell culture

All cell lines were grown at 37°C in a humidified incubator containing 5% CO_2_.

U2OS, HeLa, and SAOS cells were purchased from the American Type Culture Collection (ATCC) and cultured in Dulbecco’s modified eagle’s medium (DMEM) GlutaMAX^TM^ (Gibco) supplemented with 10% fetal bovine serum (FBS) (Gibco) and antibiotics. DAOY were a kind gift of Dr A. Di Giannatale and were cultured in MEM Medium (Gibco), supplemented with 10% FBS (Gibco) and antibiotics. ONS76 cells were purchased from Accegen and cultured in RPMI-1640 Medium (Gibco), supplemented with 10% FBS (Gibco) and antibiotics. UW228 were a kind gift of Dr D. Raleigh and were cultured in DMEM Medium (Gibco), supplemented with 10% FBS (Gibco) and antibiotics. HCT-116 2 × Flag-mAID-AMBRA1 cells stably infected with pTRIPZ-HA-TIR1 were a kind gift of Prof. M. Pagano and were maintained in McCoy’s 5A medium supplemented with 10% Tet System Approved FBS (Takara, Clontech Laboratories) and 1% penicillin/streptomycin/l-glutamine (Corning Life Sciences). Monoclonal DAOY-FUCCI, ONS76-FUCCI, and UW228-FUCCI cells were generated as previously described [19] and maintained in the same condition as their parental progenitors. Monoclonal DAOY-DHB-mVenus-p2a-mCherry-CDK4KTR, ONS76-DHB-mVenus-p2a-mCherry-CDK4KTR cells were generated as previously described [20] and maintained in the same condition as their parental progenitors. HeLa wt and KO for AMBRA1 were generated previously [21] and were cultured in DMEM Medium (Gibco), supplemented with 10% FBS (Gibco) and antibiotics.

### Transfections, plasmids, and siRNAs

Transient overexpression was performed using Lipofectamine 2000 or Lipofectamine 3000, depending on the experimental conditions, and according to the manufacturer’s instructions (Invitrogen). Plasmids for transient overexpression coding for AMBRA1 wt and AMBRA1-myc were constructed as previously described [22]. The HA-UBIQUITIN plasmid was used for transient overexpression of ubiquitin in HeLa cell line [22]. *Homo sapiens* cyclin D1-FLAG and cyclin D1-T286A-FLAG complementary DNAs (cDNAs) were generated as in [1]. For stable overexpression of cyclin D1/D2/D3 lentiviral pLVX-IRES-mCherry lentiviral vectors containing N-terminal Flag or Strep-tags were used, and pLVX-TREGS-GFP-3xFlag plasmid was used as a negative control. FFSS indicates a tandem 2 × Flag–2 × Strep tag.

For stable overexpression of cyclin D1 in the HeLa cell line, lentiviral pTripz-FFSS-CCND1 was used, and pLVX-GFP-3xFlag plasmid was used as negative control. To generate different stable FUCCI CA cell lines, the following plasmids were used in a 1:4 ratio: piggyBac-CMV-HA-tag PBase (a gift from Tiberi lab) and FUCCI CA (Addgene #153521 [19], gift from Atsushi Miyawaki). The following plasmids were used to generate the ONS76 shCTR and shp53 stable cell line: pLKO.1- puro – CMV - TurboG (Sigma–Aldrich) and pLKO.1 - puro - shp53 [23] (Addgene #19119), a gift from Bob Weinberg.

Small interfering RNA (siRNA) transfections were performed using Lipofectamine RNAiMAX (Invitrogen) according to the manufacturer’s instructions. RNA interference was performed using the oligonucleotides as previously described [1] MISSION® siRNA Universal Negative Control #1 (Sigma) was used as siRNA control. Employed siRNA sequences are as follows:

*siAMBRA1*-5′UTR: 5′-GGACAACUUACAAGGACCU-3′
*siAMBRA1*: 5′-GAGUAGAACUGCCGGAUAG-3′;
*siCCND1 – 3′UTR*: 5′- GCGUGUAGCUAUGGAAGUU-3′
*siCCND2:* 5′- GAUCGCAACUGGAAGUGUG-3′
*siCCND3:* 5′- UGCGGAAGAUGCUGGCUUA -3′
*siCDKN1A:* 5′-CUGUCACAGGCGGUUAUGA-3′
*siCDKN1B:* 5′- ACGUAAACAGCUCGAAUUA-3′
*siCDKN1A-3′UTR:* 5′-CGCTCTACATCTTCTGCCTTA-3′
*siCDKN1B-3′UTR:* 5′-GTAGGATAAGTGAAATGGATA-3′
*siDDB1*: 5′-GCUGAGUGCUUGACAUACCUUGAUA-3′
*siSKP2:* 5′ *-*GCUGCGCAUCCGCAGUAGUU-3′
*siFEN1*: 5′ *-*GATGCCTCTATGAGCATTTAT-3′
*siCDT2 #1*: 5′ *-*CTGGTGAACTTAAACTTGTTA-3′
*siCDT2 #2*: 5′ *-*GCTCCCAATATGGAACATGTA-3′

### Virus production and infection

For lentiviral production, vectors were produced by transfecting human embryonic kidney (HEK) 293T cells with the Fugene (Promega) in accordance with the manufacturer’s instructions. After removing cell debris with a filter, the lentiviruses contained in the supernatant were precipitated using a Lenti-X concentrator (TakaraBio, Cat. No. 631231) and resuspended in phosphate buffered saline (PBS). Depending on the experimental condition, cells were plated at low confluency (20%–30%) and then transduced with different amounts of the resuspended viral preparation in full medium. After 24 h, medium was removed and replaced by fresh medium, and cells were then maintained in culture for downstream analyses.

### *In vitro* ubiquitination

C-terminal 3 × Flag-tagged AMBRA1 and p21 were *in vitro* translated using the TNT T7 Quick Coupled Transcription/Translation System (Promega) according to the manufacturer’s recommendations. *In vitro* ubiquitylation reaction was carried out in buffer containing 50 mM HEPES, pH 8.0, 50 mM NaCl, 1 mM DTT, 10 mM MgATP, 100 nM E1 UBE1 (R&D Systems), 1 μM UBC H5C (R&D Systems), 2.5 μM CRL4-DDB1 (R&D Systems), 0.2 nM okadaic acid, 0.2 μM NaV, and 100 μM HA-ubiquitin (R&D Systems). Reactions were incubated at 30°C for the indicated times and then terminated by adding NuPAGE LDS sample buffer (Thermo Fisher Scientific), followed by a 5-min incubation at 95°C. Samples were resolved by sodium dodecyl sulfate–polyacrylamide gel electrophoresis (SDS–PAGE) and immunoblotted as indicated.

### Antibodies

Primary antibodies used for immunoblot (IB), immunoprecipitation (IP), immunohistochemistry (IHC), proximity ligation assay (PLA), and immunofluorescence (IF) were: α-TUBULIN (GeneTex GTX628802 - IB 1:10 000), β-actin (Sigma A2066 - IB 1:5000), Myc (Santa Cruz sc-40 - IB 1:1000, IP 500 ng), HSP90 (Santa Cruz sc13119 IB -1:10 000), AMBRA1 (Millipore ABC131 - IB 1:1000), AMBRA1 (G-6) (Santa Cruz sc-398204 - IB 1:1000), AMBRA1 (Novus 26190002 - 1:100), cyclin D1 (Cell Signaling 2978 - IB 1:1000), cyclin D1 (Abcam 16663 - IF 1:300 / IP 1:100), cyclin D2 (Cell Signaling - 3741 IB 1:1000), cyclin D2 (Santa Cruz sc-452 - IF 1:50), cyclin D3 (Thermo Fisher Scientific - MA5-12717 WB 1:1000), cyclin E2 (Cell Signaling 4132 - IB 1:1000), p21^Waf1/Cip1^ (Cell Signaling 2947 - IB 1:1000 / IF 1:100 / IHC 1:100, IP 250 ng), p27^Kip1 (^Cell Signaling 3686 - IB 1:1000, IP 250 ng), p27^Kip1^ (Santa Cruz sc-528 - 1:100), Ki67 (Abcam 15 580 - IF 1:200), HA (Y11) (Santa Cruz sc-805 - 1:500), CUL1-NEDD8 (Cell Signaling 4995 - IB 1:1000), PARP/cleaved-PARP (Abcam ab32138 - IB 1:1000), pRb 780 (Cell Signaling 8307 - IB 1:1000), pRb 807/811 (Cell Signaling 9308 - IB 1:1000), Cdt2 (Abcam ab72264 IB - 1:500), FEN1 (Novus NB100-150 PLA - 1:100), Rb (Cell Signaling 9313 IB - 1:1000), CDK4 (Cell Signaling 12790 - IB 1:1000), CDK4 (Santa Cruz sc-23896 - PLA 1:50), CDK2 (Cell Signaling 2546 - IB 1:1000), CDK2 (Santa Cruz sc-6248 - PLA 1:50), DDB1 (Novus NBP1-33061 - IB 1:1000), DDB1 (BD Biosciences 612 488 - PLA 1:100), PCNA (Santa Cruz sc-56 - IB 1:1000), PCNA (Abcam ab18197 PLA - 1:100), Chk1 (Santa Cruz sc-8408 - IB 1:1000), Chk1 (Ser345) (Cell Signaling 2341 - IB 1:1000), H2AX p-S139 (Merck Millipore 05-636 - IF 1:500), H2AX p-S139 (Abcam ab22551 - IB 1:500), CDK2 pT160 (Cell Signaling 2561 - IB 1:500), ATM (Abcam ab2618 - IB 1:1000), ATM pS1981 (Abcam ab81292 - IB 1:5000), p53 (Santa Cruz sc-126 - IB 1:500), UBIQUITIN (Santa Cruz sc-8017 - IB 1:500), SKP2 (Santa Cruz sc-7164 - IB 1:1000).

### Real time PCR (qRT-PCR)

RNA from cell lines was isolated by using NucleoSpin® RNA (MACHERY-NAGEL). First-strand cDNA was generated by using the GoScript Reverse Transcription System (Promega). Real-time PCR (RT-PCR) was performed using the iTAQ universal SYBR Green Supermix (Bio-Rad) or SYBR-Green (Applied Byosystems) on ViiA 7 Real-Time PCR System (Applied Biosystems) and QuantStudio 12K Flex (Applied Biosystems).

All reactions were run as triplicates. The resulting data were analyzed by t ViiA™ 7 Software. The fold changes in messenger RNA (mRNA) levels were determined relative to a control after normalizing to an internal standard as reported. The primers used in RT-PCR are the following:

*ACTIN* FW-CTGGGTATGGAATCCTGTGG / RV-GTACTTGCGCTCAGGAGGAG,
*CCND1* FW- CTGGCCATGAACTACCTGGA / RV- CTCCGCCTCTGGCATTTTGG,
*CCND2* FW- CACCGACTTTAAGTTTGCCA / RV- TTGGTGATCTTAGCCAGCAG

### Immunoblot analysis

Cell lysates were prepared using RIPA buffer or whole-cell lysis buffer (50 mM Tris–HCl, pH 6.8, 10% glycerol, and 2% SDS) for 5 min at 95°C with agitation at 1000 rpm. All lysis buffers were added with 1× protease and phosphatase inhibitors (Sigma–Aldrich). Protein extracts were quantified using the DC protein assay (Bio-Rad), and denatured in NuPAGE® LDS Sample Buffer (Life technologies). Then, protein extracts were subjected to SDS–PAGE and transferred to polyvinylidene fluoride (PVDF) membranes using a 25 mM Tris, 192 mM glycine wet electroblotting Trans-Blot Turbo system (Bio-Rad Laboratories) or an iBlot 2 Dry Blotting system (Thermo Fisher). The membranes were blocked using 5% (w/v) dry milk in PBS-Tween-20 (0.5% vol/vol) and probed with the indicated primary antibodies in blocking solution overnight at 4°C, followed by incubation in secondary horseradish-peroxidase (HRP)-conjugated antibodies (Bio-Rad) (1:5000) for 1 h at RT. Secondary antibody detection was performed using Amersham ECL Prime (GE Healthcare) or Immobilon ECL Ultra Western HRP Substrate (Merck Millipore IBULS0100), and the signal was acquired using either the Invitrogen iBright CL1500 or the MP900E (Colenta) developer. Densitometric levels were quantified using ImageJ.

### Co-immunoprecipitation

Cells were lysed in a buffer composed of 150 mM NaCl, 0,3% CHAPS, 40 mM pH 7.5 HEPES, 2 mM ethylenediaminetetraacetic acid (EDTA). Lysates (0.5–1 mg) were then incubated at 4°C for 30 min. Samples were precleared from unspecific binding with 10 μl of magnetic Dynabeads Protein G for 1 h at 4°C. Depending on experimental conditions, equal amounts of protein were incubated with 250 ng of monoclonal anti-p21^Waf1/Cip1^ (Cell Signaling 2947) or anti-p27^Kip1^ (Cell Signaling 3686), or 500 ng anti-Myc tag (Santa Cruz sc-40) overnight at 4°C, followed by 60-min incubation with 10 μl of magnetic Dynabeads Protein G (Invitrogen).

To detect ubiquitination levels of p21^Waf1/Cip1^ and p27^Kip1^, HeLa cells overexpressing HA-tagged ubiquitin were lysed in 150 mM NaCl, 0.4% NP-40, 10% glycerol, 1 mM ethylenediaminetetraacetic acid (EDTA), 1 mM ethyleneglycol- bis(β-aminoethyl)-N,N,Nʹ,Nʹ-tetraacetic acid (EGTA), 3 mM MgCl_2_, 50 mM pH 7.4 HEPES. Lysates were then incubated at 4°C for 60 min, and 1% SDS was added to the final volume recovered. This was followed by a 5-min incubation at 90°C and 300 rpm. The samples were then diluted seven-fold using the initial lysis buffer, and equal amounts of protein were incubated overnight at 4°C with anti-p21^Waf1/Cip1^ or anti-p27^Kip1^. This was followed by a 60-min incubation with 10 μl of magnetic Dynabeads Protein G (Invitrogen).

For mass spectrometry analysis, HeLa wt and KO for AMBRA1 were overexpressed with either Flag p21 WT (Addgene #16240) or pMSCV-FLAG-2xSTREP-CDKN1B-Puro (Addgene #172611). For cell lysis buffer solution composed of 1% NP40, 50 mM, pH 7.5 HEPES, 150 mM NaCl, protease inhibitor (Sigma), and phosphatase inhibitors NaF 5mM, Na3VO4 5mM, and beta-glycerophosphate 5 mM. Lysates were then incubated at 4°C for 60 min. Flagged recombinant peptides were immunoprecipitated with Pierce™Anti-DYKDDDDK Magnetic Agarose (Product No. A36797) and then eluted with Pierce™ 3× DYKDDDDK Peptide (Product No. 36805), according to manufacturer’s instructions.

### LC-MS/MS analysis

Instruments for LC-MS/MS analysis consisted of a NanoLC 1200 coupled via a nano-electrospray ionization source to the quadrupole-based Q Exactive HF benchtop mass spectrometer. For the chromatographic separation, a binary buffer system consisting of solution A (0.1% formic acid) and solution B (80% acetonitrile, 0.1% formic acid) was used. The peptides were separated according to their hydrophobicity on an analytical column (75 μm) in-house packed with C18-AQ 1.9 μm C18 resin with a gradient of 7%–32% solvent B in 45 min, 32%–45% B in 5 min, 45%–95% B in 3 min, 95%–5% B in 5 min at a flow rate of 300 nl/min. MS data acquisition was performed in DIA (Data Independent Acquisition) mode using 32 variable windows covering a mass range of 300–1650 m/z. The resolution was set to 60 000 for MS1 and 30 000 for MS2. The AGC was 3e6 in both MS1 and MS2, with a maximum injection time of 60 ms in MS1 and 54 ms in MS2. NCE were set to 25%, 27.5%, and 30%.

All acquired raw files were processed using Spectronaut 18 software. For protein assignment, spectra were correlated with the Human data base (v. 2023). Searches were performed with tryptic specifications and default settings for mass tolerances for both MS and MS/MS spectra.

The other parameters were set as follow:

- Fixed modifications: Carbamidomethyl (C)
- Variable modifications: Oxidation, Acetyl (N-term)
- Digestion: Trypsin, Lys-C
- Min. peptide length = 7 Da
- Max. peptide mass = 470 Da
- False discovery rate (FDR) for proteins and peptide-spectrum = 1%

The Perseus software (1.6.2.3) was used to logarithmize, group, and filter [after filter] the protein abundance. FDR corrected *t*-test analysis were performed, setting FDR = 0.05, for proteins with differences. ANOVA *q*-value or Log_2_ Difference≥ ± 0.5 and *q*-value < 0.05 were considered significantly enriched (see “Significantly deregulated” column in the Excel file).

### Flow cytometry and FACS sorting

Stable cell lines expressing the FUCCI CA plasmid reporter were washed with PBS-1X, trypsinized, and collected in DMEM without phenol red (Gibco, Thermo Fisher Scientific). Cells were filtered using a 40 µm Cell Strainer (Falcon) and kept on ice. Cell cycle analysis and sorting of cell cycle phase subpopulations were carried out using the FACS Celesta (BD Bioscience) with the FITC filter to detect cells in S phase. Cells in G1 were identified by RFP, and cells showing a double-positive signal (FITC+/RFP+) were considered to be in the G2/M phase (Supplementary Information). For cell sorting, a FACS Aria cell sorter was used, and at least 1 × 10^5^ cells for each cell cycle phase were sorted. Cells were collected by centrifugation, and the pellet was used for IB sample preparation.

### PLA—proximity ligation assay

DAOY cells were plated in fibronectin-coated 13-mm slides. Cells were then transiently transfected for AMBRA1 interference expression for 48 h before being fixed with 4% PFA. Coverslips were then processed with the Duolink PLAs Kit (Sigma–Aldrich) according to the manufacturer’s instructions and, finally, stained using Duolink In Situ Mounting Media with DAPI for 15 min. Samples were mounted on the slide, and images were acquired using a Zeiss Confocal Microscope and CellDiscoverer7 at 20× magnification. Foci detection was performed through a customized pipeline in Arivis Vision4D software, version 4.1.1.

### Annexin V staining

Cells were washed twice with cold PBS and then resuspended in 1× Annexin V Binding Buffer (BD Pharmigen #51-55121E). 1 × 10^5^ cells were transferred into 5-ml round-bottom tubes. Five microliters of APC Annexin V (BD Pharmigen #550475) were added to each culture tube, and cells were incubated for 15 min at RT in the dark. The tubes were analyzed by flow cytometry immediately after.

### Cell viability assay

To obtain the total cell count, cells were stained with CellTracker 488 (Invitrogen) for 30 min at 37 °C and analyzed using a Celigo imaging cytometer (Nexcelom).

### UV irradiation and pharmacological treatments

HeLa wt and KO for AMBRA1, DAOY, and ONS76 cells were exposed to UV radiation of either 20, 40, or 60 J/m^2^, according to experimental conditions using an UV Stratalinker 2400.

For protein stability evaluation, cells were treated with 100 μg/ml CHX (cycloheximide - SIGMA C4859) alone or in combination with 5 μM MG132 (SIGMA M7449) for the indicated time points. NEDD-8 activating enzyme inhibitor, MLN4924, for Cullins inhibition was used in DAOY, ONS76, HeLa wt, and KO for AMBRA1 for 4 h with 2,5 μM. Cells were treated with AZD7762 (SML0350 Sigma) 200 nM and FEN1i 10 µM. MB cell lines were treated with either abemaciclib or palbociclib for 24 h at the indicated doses. HCT-116 2× Flag-mAID-AMBRA1 cell line was treated with doxycyclin 1 µg/ml (D9891, Sigma), Auxin 0,5 mM (I5148, Sigma), and MS28 6 µM (Xiong, Y. *et al*., 2022) for the time points indicated in figure legends.

### Quantitative image-based cytometry

For immunocytochemistry, cells were grown on 96-well (Greiner Screenstar 655866) 24 h before the transfection. Forty-eight hours after the initial silencing, cells were incubated with 10 µM EdU for 30 min at 37 °C before were fixed in 4% paraformaldehyde in PBS for 15 min and washed three times in PBS. Permeabilization was performed in PBS plus 0.5% Triton X-100 for 5 min at room temperature (RT), followed by 30 min of blocking in the blocking buffer [PBS plus 5% FBS, 1% bovine serum albumin (BSA), and 0.2% Triton X-100]. Click-iT EdU Imaging Kits (Invitrogen) were used for EdU detection according to the manufacturer’s instructions. Cells were incubated with primary antibodies in a blocking buffer for 2 h at RT and washed three times in PBS plus 0.1% Triton X-100. Then, cells were incubated with the appropriate secondary antibodies conjugated to Alexa Fluor 488 or Alexa Fluor 568 (Life Technologies) diluted in a blocking buffer for 45 min at RT. DNA was stained using Hoechst 33342 (Thermo Fisher Scientific, H3570, 1:1000 in PBS) 10 min at RT. Automated multichannel wide-field microscopy for high-content imaging and quantitative image-based cytometry (QIBC) was performed using CellDiscoverer 7 (Zeiss) with a 20× magnification objective. After background subtraction, cells were segmented for the cytoplasmic and nuclear areas with Arivis software using a customized pipeline implemented with the cellpose algorithm. For CDK2 and CDK4/6 activity analysis, cells were segmented using the same pipeline. CDK4/6 activity correction based on the activity of the CDK2 reporter was performed as described previously (Yang *et al*., 2020). For each condition, 6 × 6 tiles of three independent experiments were acquired, and at least 1000 cells per replicate were processed using Zen 2.6 (Blue Edition) software (Zeiss). Scatter plots and bubble plots were generated with the ggplot2 package in R.

### DNA fiber analysis

Cell cultures transfected with siRNA and/or treated with different drugs were pulse-labeled with 25 μM of CldU (Sigma–Aldrich) for 20 min, followed by a gentle wash with fresh pre-warmed media and the second pulse of 250 μM of IdU (Sigma–Aldrich) for 20 min. Labeled cells were harvested, and DNA fiber spreads prepared as previously described [1]. For every single experimental condition, five slides were stretched, and 2–3 slides for each condition were stained. CldU was detected first with the rat anti-BrdU antibody (Serotec, OBT0030) and IdU with the mouse anti-BrdU antibody (Becton Dickinson, 347580). Secondary antibodies were DyLight 550 anti-rat (Thermo Fisher Scientific) and Alexa Fluor 488 anti-mouse (Invitrogen), respectively. Images of well-spread DNA fibers were acquired using a LSM800 confocal microscope (Carl Zeiss) and a Plan-Apochromat 63×/1.4 N.A. oil immersion objective (Carl Zeiss). Images were acquired semi-automatically using the software’s autofocus and tile-array modes. Double-labeled replication forks were analyzed manually using LSM ZEN software. Between 100 and 250 replication forks were scored for each slide, and fork measurements for all slides for the same experimental condition were pooled together. At least one additional independent experiment was performed, and, if the experiments did not differ statistically, the total number of DNA fibers from all experiments is presented.

### S1 nuclease assay for DNA fibers

Cell cultures in 6-cm dishes transfected with siRNA were pulse-labeled with 250 μM of ldU (Sigma–Aldrich) for 60 min, followed by a gentle wash with PBS. Cytoskeletal disruption was achieved using 0.5 ml of fresh CSK100 (100 mM NaCl, 10 mM MOPS, 3 mM MgCl_2_, 300 mM sucrose, 0.5% Triton X-100) for 10 min at RT. After a careful wash with PBS and equilibration using the S1 nuclease buffer (30 mM sodium acetate, 10 mM zinc acetate, 5% glycerol, 50 mM NaCl, pH 4.6), the cells were treated with 20 U/ml S1 nuclease for 30 min at 37°C. After S1 nuclease removal, nuclei were harvested using a cell scraper and 0.5 ml of 0.1% BSA in PBS. Nuclei were kept on ice for subsequent DNA fiber spreads (adapted protocol from Quinet *et al*. 2017).

### Live imaging

For U2OS-FUCCI time-lapse imaging, cells were plated at a low density (~10 000 cells per well) and imaged in 96-well plates in DMEM inside a heated 37°C chamber with 5% CO_2_. Images were taken every 30 min with a 20×/0.5 NA air objective using a Zeiss Cell Discoverer 7. For cell tracking, ImageJ plugin TrackMate [24] with StarDist segmentation was used.

### Histology and immunohistochemistry

IF analysis of tissue sections of the Ambra1/Nestin-Cre mouse model were carried out on cryoembedded embryos (OCT). Before embedding, the embryos and pups’ brains were fixed for 24 h in 4% formaldehyde in PBS, then cryoprotected with a 30% sucrose solution in PBS for 48 h. After sinking, tissues were enclosed in a 1:1 mixture of OCT (Thermo Fisher) and 30% sucrose/PBS solution, embedded in cryomolds that were pre-chilled in an isopentane bath on dry ice, and stored at −80°C. Lastly, tissues were cut using a Leica CM3050S cryostat and placed on SuperFrost Plus slides (Thermo Fisher). For IF, sections were washed and rehydrated with PBS, permeabilized, and blocked with 0.3% Triton X in Protein Block (Abcam) solution. The slides were incubated with primary antibodies in Protein Block overnight at 4°C in a humidified chamber. Sections were then washed three times in PBS and incubated with the appropriate secondary antibodies conjugated to Alexa Fluor 488 or Alexa Fluor 568 (Life Technologies) diluted in Protein Block for 2 h at RT. Eventually, sections were stained with Hoechst 33342 for 10 min at RT. The slides were mounted with Fluoromount (Sigma F4680). IF images were taken with Hamamatsu Nanozoomer S60 or Confocal Microscope Olympus FV1000. For H/E of MB-SHH patients, sections were then dipped in Gill’s Hematoxylin No. 2 Solution (Bio-Optica, Cat. No. 05-06014/L) for 30 s. Sections were washed in ddH_2_O, followed by 0.5% alcoholic eosin (Diapath, Cat. No. C0353). Sections were dehydrated with one 10-s wash in 90% ethanol and three 10-s washes in 100% ethanol. Lastly, sections were immersed in Diasolv (Diapath, Cat. No. H0315) for three times.

All samples of human tissue research in this study were collected at the Bambino Gesù Children’s Hospital in Rome, with Institutional Review Board approval. These clinical MB specimens were examined and diagnosed by pathologists. Tissue sections of clinical specimens were stained with antibodies against AMBRA1, cyclin D1, and p21^Waf1/Cip1^. The analyses were assessed by an experienced pathologist (S.R.), blinded to the sample. Digital images were acquired by a Hamamatsu NDP Nanozoomer digital pathology slide scanner.

### Senescence-associated β-galactosidase assay

Cellular senescence was assessed 48 h post-transfection using the Senescence β-Galactosidase Staining Kit (Cell Signaling Technology, #9860) following the manufacturer’s protocol. ONS76 cells were washed with PBS and fixed in 1× Fixative Solution for 10–15 min at RT. Cells were subsequently incubated overnight (16–24 h) at 37°C in a CO_2_-free dry incubator with senescence-associated β-galactosidase (SA-β-gal) Staining Solution at pH 6.0. The appearance of the characteristic blue perinuclear precipitate was assessed by brightfield microscopy. Senescence was quantified by scoring the percentage of SA-β-gal-positive cells across a minimum of 50 cells per biological replicate.

### Analyses of publicly available transcriptomics data

We used the R2 genomics platform (http://r2.amc.nl) to identify suitable transcriptomics datasets with molecular and histological subtypes available for MB. We selected the following datasets: Pomeroy (204 samples, MAS5.0-normalized, u133a chip) and Cavalli (763 samples, rma_sketch normalized, hugene11t chip). For each of them, we download log_2_-transformed and normalized data through the data grabber function for each subgroup (normal cerebellum, SHH) and subtype (SHH-α, β, γ, and δ) for AMBRA1 (52731_at probe) expression levels. We employed a parametric Welch’s *t*-test to assess the significance of the differences in expression levels of the same gene in the different subgroups and subtypes.

### DepMap co-occurrence analysis

Public 22Q2 CERES-corrected gene effects of CRISPR KO were downloaded from the DepMap portal (https://depmap.org/portal/). Correlation analysis was performed, and the top 500 genes were isolated for AMBRA1 co-dependencies. Following pathway analysis performed through ConsensusPathDB-human and the top 15 Reactome pathways were plotted.

### Kaplan–Meier survival

Survival analysis was generated using the dataset GSE85217 (Cavalli). AMBRA1 gene expression levels were expressed as a binary variable using the cut-off value generated by the Kaplan Scan online algorithm (https://hgserver1.amc.nl/cgi-bin/r2/main.cgi?option=kaplanscan1). The overall survival right-censored at 5 years, was estimated using the Kaplan–Meier method, and *P*-values were assessed using the log-rank test to express statistical differences in overall survival among patients in MB-SHH.

### Statistical analysis and data reproducibility

Statistical analyses were performed with Prism software (GraphPad Software) or with R environment. All statistical parameters, including the exact value of *n*, type of replicates, the statistical test, error bars, and significance, are reported in all associated figure legends. Unless otherwise stated all experimental findings were verified in ≥3 independent experiments.

No statistical methods were used to pre-determine the sample size. The experiments were not randomized, and the investigators were not blinded to allocation during experiments and outcome assessment.

## Results

### AMBRA1 regulates the stability of p27 and p21 in MB-SHH cell lines

**Figure 1.**
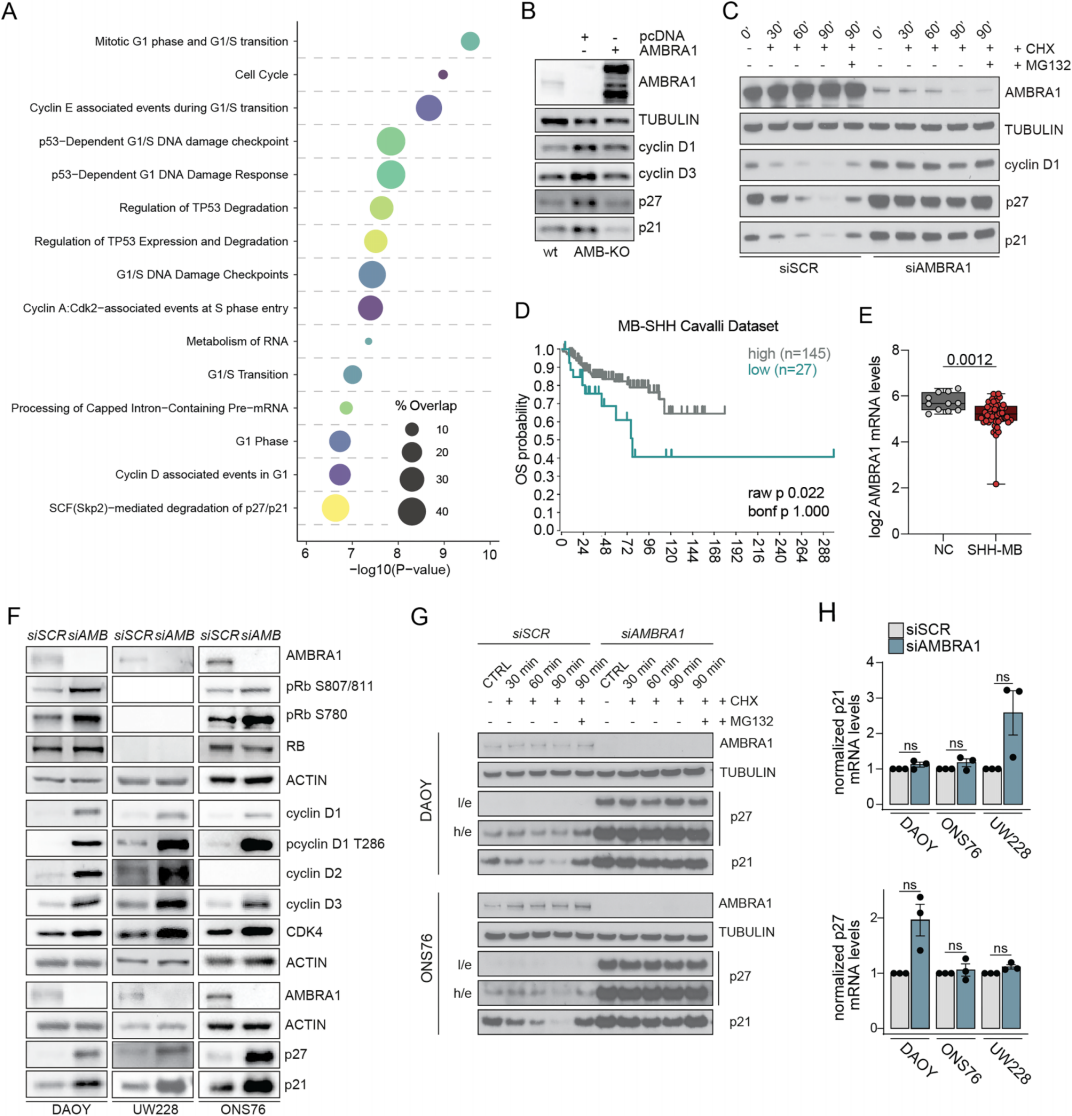
AMBRA1 regulates p21 and p27 stability in MB-SHH cell lines. (**A**) Bubble plot of pathway enrichment analysis for AMBRA1 top 500 co-dependencies from DepMap CRISPR KO database. (**B**) IB of the indicated proteins in wt or *AMBRA1*-KO HeLa cells, reconstituted or not with a plasmid overexpressing AMBRA1 (*n* = 3). (**C**) IB of the indicated proteins in HeLa cells depleted for AMBRA1 with siRNA and treated with 100 µg/ml CHX and/or 10 µM MG132 for the indicated time points (*n* = 3). (**D**) Kaplan–Meier analysis for MB-SHH patients according to AMBRA1 expression levels in Cavalli dataset. Low AMBRA1 *n* = 27; High AMBRA1 *n* = 145. Expression cut-off 239 500. (**E**) Boxplot for mRNA log_2_ expression of AMBRA1 derived from the publicly available dataset Pomeroy (204 patients, MAS 5.0 normalized, u133a platform), presented as mean value ± SD. NC, normal cerebellum = 11; MB-SHH = 54. (**F**) IB for the indicated proteins in three MB-SHH cell lines: DAOY, UW228, and ONS76 in control or AMBRA1-depleted conditions with siRNA. (**G**) IB of the indicated proteins in DAOY and ONS76 cells depleted for AMBRA1 with siRNA and treated as in panel (C) (*n* = 4). (**H**) Barplot of quantitative PCR with qRT‒PCR of the indicated genes in the three MB-SHH cell lines. Data were analyzed using the log-rank test (D), Welch’s two-tailed unpaired *t*-test (E), and unpaired Student *t*-test (H).

To gain deeper insight into the impact of AMBRA1 loss on cellular physiology, we analyzed data from the Cancer Dependency Map Portal [25], identifying genes that exhibit co-dependencies with AMBRA1 knockout (AMBRA1-KO). GeneEffect correlations revealed a potential involvement of AMBRA1 in the degradation of p21 and p27 (Fig. 1A). Given their significance, we assessed p21 and p27 protein levels in AMBRA1-KO cells, using cyclin D1 and D3 as controls, both before and after reconstitution of AMBRA1. Strikingly, AMBRA1-KO led to the accumulation of p21 and p27, a phenotype reversed by AMBRA1 reconstitution (Fig. 1B). To assess whether the elevated p21 and p27 levels observed upon AMBRA1 depletion resulted from defective proteasomal degradation, we combined inhibition of *de novo* protein synthesis with CHX and proteasome inhibition with MG132, which confirmed a pronounced stabilization of these proteins (Fig. 1C).

We have previously shown that AMBRA1 is differentially expressed across the molecular subtypes of MB, with the highest expression in Group 3 and WNT, and the lowest in SHH [13], making MB an ideal model to study the effect of different expression levels of AMBRA1 in cell pathophysiology. Analysis of available MB-SHH datasets (deposited at http://r2.amc.nl) revealed that AMBRA1 low expression correlates with reduced survival and that its mRNA levels are downregulated in this tumor context compared to normal cerebellar cells (Fig. 1D and E). The intricate shaping of the cerebellum relies on a meticulous regulation of the cell cycle, directing the fate decisions of individual cells during differentiation and stem cell functions [26]. MB, as an embryonal pediatric cancer, stems from aberrant cerebellar development. To investigate the impact of *Ambra1* absence on cerebellar development *in vivo*, an *Ambra1^flox/flox^*/*Nestin-Cre* mouse model was used. Cre recombinase activation led to *Ambra1* deletion, resulting in a marked increase in cerebellar anlage proliferation at E13.5 (Supplementary Fig. S1A) and correlating with elevated expression of D-type cyclins (Supplementary Fig. S1B). Later in development (E18.5), the newly formed cerebellum displayed a more proliferative external granule layer (Supplementary Fig. S1C), a region populated with granule cell precursors. Indeed, *Ambra1* depletion resulted in high levels of cyclin D1, p21, and phospho-Rb (S807–811) in this region (Supplementary Fig. S1D–F). Altogether, these results underscore the critical role of AMBRA1 in normal cerebellar development and suggest its potential significance in MB.

To elucidate the molecular processes regulated by AMBRA1 in MB-SHH, we used three available cell lines isolated from this tumor subtype, DAOY, UW228, and ONS76 [27]. In all three cell lines, the reduction of AMBRA1 resulted in the accumulation of cyclins D1–3, together with higher CDK4, p21, and p27 (Fig. 1F). Relevant to the regulation of the cell cycle, in an AMBRA1-depleted condition, we observed an increase in phospho-Rb (S807–811) in DAOY and ONS76 cell lines. In the UW228 cell line, there were no detectable levels of either total or phosphorylated Rb, suggesting aberrant Rb loss. The augmented stability of p21 and p27 upon AMBRA1 depletion in these cell lines was confirmed by a time-course experiment using CHX and MG132 (Fig. 1G and Supplementary Fig. S1G and H). To rule out the possibility of upregulation of p21 and p27 transcription in AMBRA1-depleted cells, we analyzed transcript levels by RT-PCR. The results showed that in the absence of AMBRA1, the expression levels of p21 and p27 transcripts did not change significantly across the three cell lines (Fig. 1H). Collectively, these findings underscore the regulatory role of AMBRA1 in modulating p21 and p27 protein turnover, in a manner that parallels its modulation of D-type cyclins [1, 9].

### The role of cyclin D in AMBRA1-mediated degradation of p21 and p27

**Figure 2.**
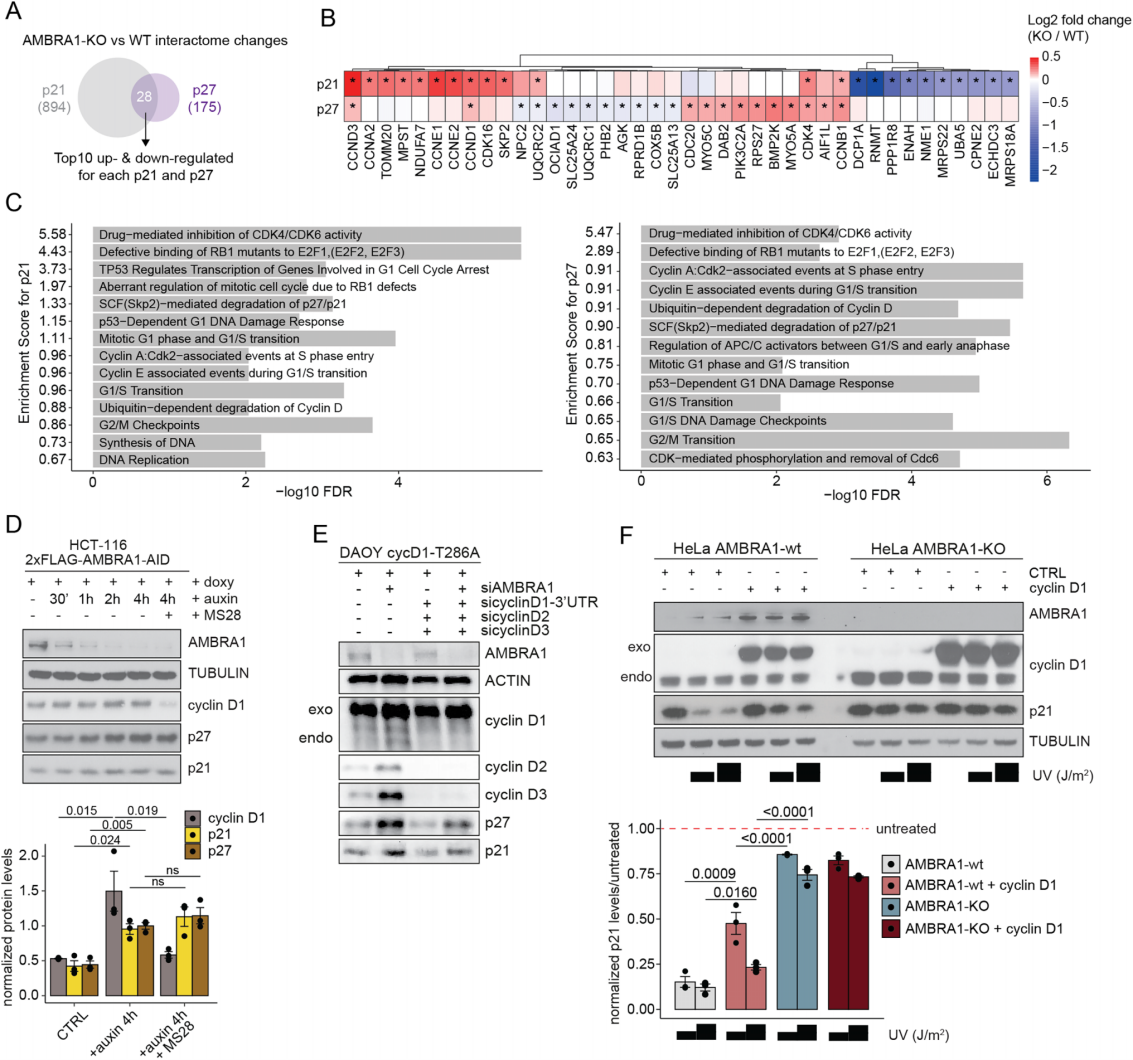
AMBRA1 mediates p21 and p27 stability and the contribution of cyclin D1. (**A**) Venn diagram for shared p21 and p27 interactors in HeLa cells affected by AMBRA1 KO, obtained by LC-MS (*n* = 4). (**B**) Heatmap of top10 up- and down-regulated interactions from proteomics analysis in wt and *AMBRA1*-KO HeLa cells overexpressed with either p21 or p27 Flag-tagged plasmids. Asterisks for proteins indicate significant differences to wt (adjusted *P* value < .05, FDR-adjusted *P-*values from two-tailed Student’s *t*-test). Bold text indicates proteins shared in both interactomes. (**C**) Top 14 most enriched cell cycle-related biological processes (FDR < 0.01) from STRING (Functional Enrichment Analysis) affected by *AMBRA1*-KO in p21 (left) and p27 (right) interactomes. (**D**) Top: IB for the indicated proteins of whole-cell extracts from 2× Flag-mAID-AMBRA1 HCT-116 cells pre-treated with either DMSO or doxycycline for 12 h and exposed to a combination of auxin and MS28 (cyclin D1 PROTAC degrader) for the indicated amount of time (*n* = 3). Bottom: barplot for the corresponding normalized quantification of p21, p27, and cyclin D1 levels. (**E**) IB for the indicated proteins in DAOY cells constitutively overexpressing the phospho-silent mutant of cyclin D1 T286A, depleted for the indicated genes with siRNA (*n* = 3). (**F**) Top: IB of the indicated proteins in wt or *AMBRA1*-KO HeLa cells constitutively overexpressing a doxycycline-inducible form of cyclin D1. Cells were treated with either 20 or 40 J/m^2^ UV irradiation 15 min before harvesting (*n* = 3). Bottom: barplot for the corresponding over CTRL-normalized quantification of p21 levels. Unless otherwise stated data are presented as mean value ± SEM, and *n* refers to biological independent samples. (D, F) Data were analyzed using an unpaired Student *t*-test.

Depending on the complexes to which they are bound, p21 and p27 can have a dual effect in regulating G1/S transition [6, 8, 28]. To identify differential protein interactors influenced by AMBRA1 loss, we immunoprecipitated p21 and p27 in parental and AMBRA1-KO cells and performed LC-MS. In the absence of AMBRA1, there was an enrichment of p21 and p27 interacting with the complex CDK4-cyclin D, suggesting an unbalanced distribution of both p21 and p27 toward the catalytically active complexes that promote transition into the S phase (Fig. 2A and B; Supplementary Fig. S2A). The interactome shows the most significant changes in cell cycle-related pathways, with upregulation of proteins involved in the G1/S transition and the p53-dependent G1 DNA damage response (DDR) (Fig. 2C).

Overexpression of cyclin D1 has been reported to promote stabilization of p21 in a proteasome-dependent manner by competing for p21’s proteasomal binding [29]. Consistent with this mechanism, we also observed that overexpressing the three cyclin D family members stabilizes p21 and p27 in our MB model cell line (Supplementary Fig. S2B). Since we and others have previously shown that AMBRA1 absence results in cyclin D1 accumulation [1, 9, 30], we hypothesized that AMBRA1 could affect p21 stability in a cyclin D-dependent manner. To this end, we employed a recently developed PROTAC degrader for cyclin D1, MS28 [31]. First, AMBRA1 protein degradation was induced using a Flag-tagged minimal auxin-inducible degron (2× Flag–mAID), followed by the degradation of cyclin D1. The results showed that cyclin D1 depletion did not affect the enhanced stability of p27 and p21 in the absence of AMBRA1 (Fig. 2D). To exclude that this phenotype is cell-cycle dependent, we engineered DAOY cells to constitutively express cyclin D1 (T286A), a mutant insensitive to ubiquitin-mediated degradation that cannot interact with AMBRA1 [1, 9]; then, we collectively knockdown each of the endogenous D-type cyclins alone or in combination with AMBRA1 depletion. The experiments revealed that upon AMBRA1 depletion, p21 and p27 were stabilized even in the absence of cyclin D, albeit to a lesser extent (Fig. 2E). Next, we generated cells with doxycycline-inducible cyclin D1 expression in both AMBRA1-wt and -KO cells and treated them with low dosages of UV irradiation, a well-known trigger of p21 degradation as part of the DDR [2]. IB analysis confirmed that while cyclin D1 overexpression alone reduced the degradation of p21, its effect was less pronounced than that of AMBRA1-KO. Furthermore, the combined treatment showed no additional impact beyond AMBRA1-KO alone (Fig. 2F). Importantly, the overexpression of cyclin D1 did not affect the levels of ubiquitination of p27 or p21 (Supplementary Fig. S2C).

To further delineate the potential contributions of p21 and/or p27 to cyclin D1 stabilization following AMBRA1 loss, we next examined their roles in regulating cyclin D turnover. Intriguingly, the cyclin D1 accumulation upon AMBRA1 depletion was prevented by co-depletion of p21 with AMBRA1 in both MB cell lines. In contrast, p27 silencing produced only a minor and inconsistent effect on cyclin D1 across the two cell lines (Supplementary Fig. S2D). The observed changes in protein levels under the tested experimental conditions was independent of the mRNA levels (Supplementary Fig. S2E). Overall, these results suggest the existence of an AMBRA1-dependent mechanism regulating the stability of p27 and p21, a phenomenon that occurs, at least in part, independently of cyclin D1 levels.

### AMBRA1 regulates p21 and p27 ubiquitination via CRL4^DDB1^ complex

**Figure 3.**
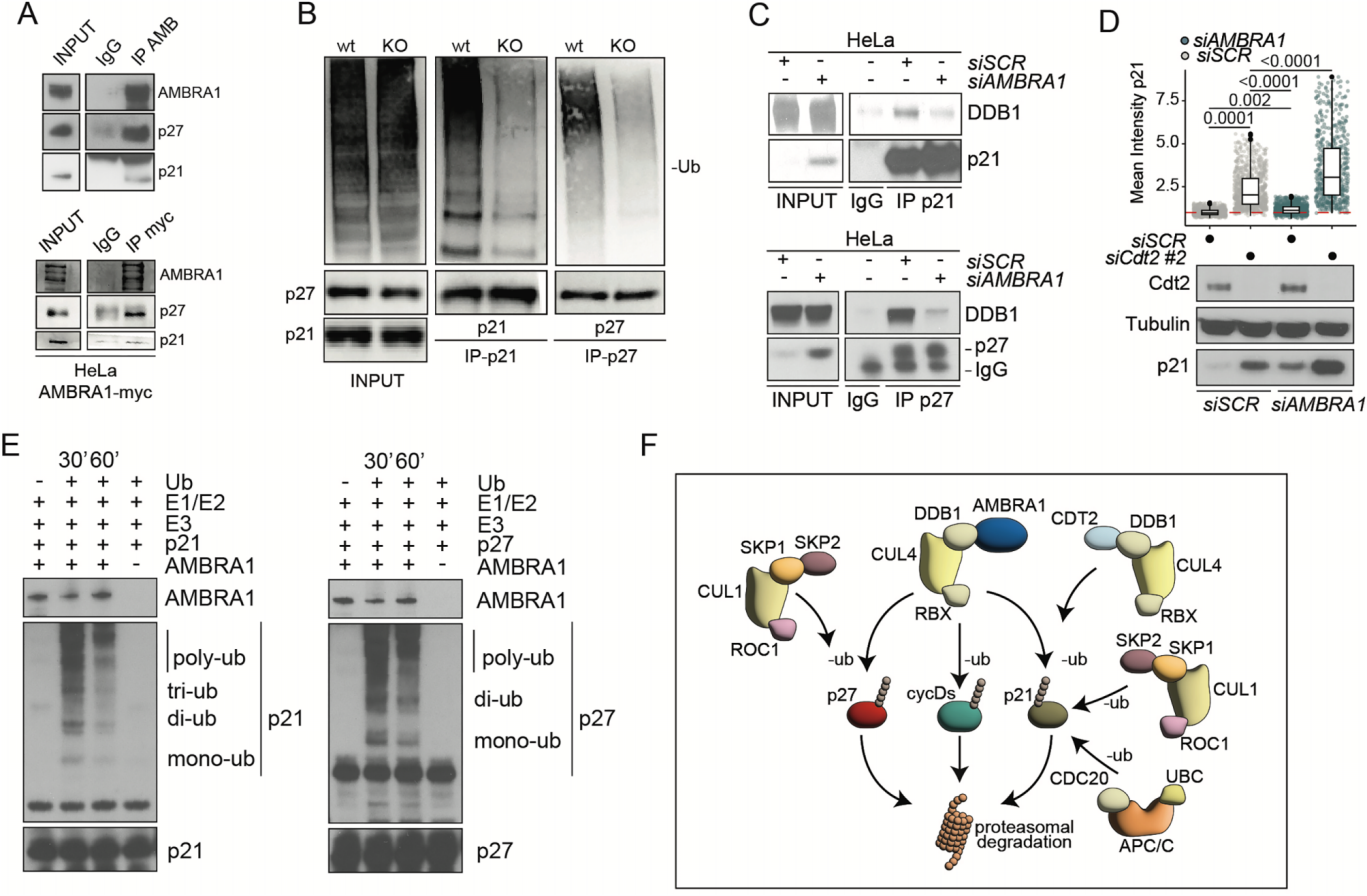
AMBRA1 mediates p21 and p27 ubiquitination. (**A**) IB of the indicated proteins for the co-IP of endogenous (top) and over-expressed (bottom) AMBRA1 (*n* = 3). (**B**) IB for the *in vivo* ubiquitination levels of p21 and p27. HeLa cells wt or KO for *AMBRA1* were transfected to overexpress a plasmid encoding for a HA-tagged form of ubiquitin and treated with 5 µM MG132 before harvesting (*n* = 3). (**C**) Co-IP of the indicated proteins (p21, top - p27, bottom) in HeLa cells depleted for AMBRA1 (*n* = 2). (**D**) Top: boxplot for QIBC (median values with IQR) analysis for the indicated proteins in DAOY cells silenced or not for the indicated genes (*n* = 1000 in three independent technical replicates). Bottom: IB of cells treated as previously indicated (*n* = 2). (**E**) IB of the indicated proteins for *in vitro* transcribed and translated AMBRA1, p21 (left), and p27 (right) were incubated in the presence of wild-type ubiquitin (Ub) and synthetic CRL4^DDB1^ complex. Reactions were stopped with Laemmli loading buffer. (**F**) Graphical representation illustrating the various pathways involved in the degradation of p21, p27, and D-type cyclins ubiquitylation and degradation. Unless otherwise stated, *n* refers to biological independent samples.

To directly assess how AMBRA1 is mechanistically involved in the regulation of p21 and p27 stability, we performed co-immunoprecipitation (Co-IP) experiments under both endogenous and semi-endogenous conditions, revealing that both p21 and p27 interact with AMBRA1 (Fig. 3A). Next, we immunoprecipitated both p21 and p27, in both MB and non-MB cell lines, to assess whether the extent of ubiquitination of these proteins is affected by the absence of AMBRA1. These experiments showed that upon proteasome inhibition, the polyubiquitination of p21 and p27 was reduced in the absence of AMBRA1 (Fig. 3B and Supplementary Fig. S3A).

Multiple E3 ligases have been found to modulate cell cycle-related proteins in a time-dependent manner [32]. Among those, CRLs represent the most prominent E3 ubiquitin ligase family and are characterized by a modular architecture. The interchangeable cullin family members (CUL1, CUL2, CUL3, CUL4A, CUL4B, and CUL5), together with their respective adaptor proteins, such as F-box proteins or DDB1, confer substrate recognition and recruitment. To test CRLs’ roles in controlling the levels of cyclin D1, p27, and p21, we treated cells with MLN4924, a small molecule that inactivates CRLs by blocking cullin neddylation [33]. Similar to what was observed upon AMBRA1 depletion in a cell-type-independent manner, inhibition of CRLs resulted in the accumulation of the above three proteins (Supplementary Fig. S3B). Furthermore, from our earlier mass spectrometry analysis, SKP2 was identified as enriched among p21 interactors upon AMBRA1-KO, ruling out that AMBRA1 could promote the SKP2-mediated degradation of p21.

This finding prompted us to investigate the specific involvement of AMBRA1 in the CRL4^DDB1^ degradative pathway. To this end, we immunoprecipitated p21 and p27 from control and AMBRA1-depleted cells, followed by an assessment of DDB1’s ability to interact with both proteins. The absence of AMBRA1 impaired DDB1-p21/-p27 interactions, suggesting AMBRA1’s direct involvement in CRL4^DDB1^ ubiquitination-mediated processes (Fig. 3C). Since the CRL4^DDB1^ complex is known to regulate p21 stability through the substrate receptor Cdt2 upon UV irradiation [2], we investigated whether AMBRA1 functions within this axis or operates via a parallel pathway. To explore this, Cdt2 and AMBRA1 were silenced individually or in combination and p21 levels were measured using QIBC and IB analysis. While depletion of either factor led to p21 upregulation, co-depletion resulted in an even greater increase in p21 levels, suggesting that AMBRA1 and Cdt2 regulate p21 through parallel mechanisms (Fig. 3D and Supplementary Fig. S3C). Finally, we performed a well-established assay to demonstrate the participation of the CRL4^DDB1^ complex in the ubiquitination of p21 and p27 [9]. Recombinant p21 or p27 served as substrates for the CRL4^DDB1^ complex and were incubated in the presence or absence of *in vitro*–translated AMBRA1. This assay demonstrated that AMBRA1 promotes the ubiquitination of both p21 and p27, indicating its role as a positive regulator of CRL4^DDB1^-mediated ubiquitination (Fig. 3E). In summary, our findings demonstrate that AMBRA1 regulates the ubiquitynation and proteasomal degradation of p21 and p27 through the E3 ligase CRL4^DDB1^. This mechanism operates alongside other established pathways contributing to p21 and p27 degradation, such as the CRL4^DDB1^–Cdt2, CUL1–SKP2, and the APC/C-mediated pathways (Fig. 3F) [3, 34, 35].

### AMBRA1 regulates G1/S transition and prevents DNA damage via cyclin D1–p21 stabilization

The initial discovery of Cip/Kip family members characterized them as nuclear proteins with a predominant function of inhibiting cyclin–CDK activity and cell-cycle progression [36]. However, building evidence has clarified their dual role: they slow down the cell cycle by inhibiting the CDK2–cyclin E/A complex while promoting the G1/S transition through the promoting the assembly and nuclear translocation of the CDK4–cyclin D complex [6]. This regulation is closely linked to the retinoblastoma protein (Rb), a central gatekeeper of the G1/S checkpoint. Hypo-phosphorylated Rb binds E2F transcription factors, preventing the expression of genes required for S-phase entry. Activated CDK4/6–cyclin D complexes phosphorylate Rb, thereby relieving its repression of E2F transcription factors and facilitating G1/S progression. By stabilizing CDK4–cyclin D, Cip/Kip proteins indirectly promote Rb phosphorylation, highlighting their nuanced role in balancing cell cycle inhibition with the necessary advance past the restriction point [7].

**Figure 4.**
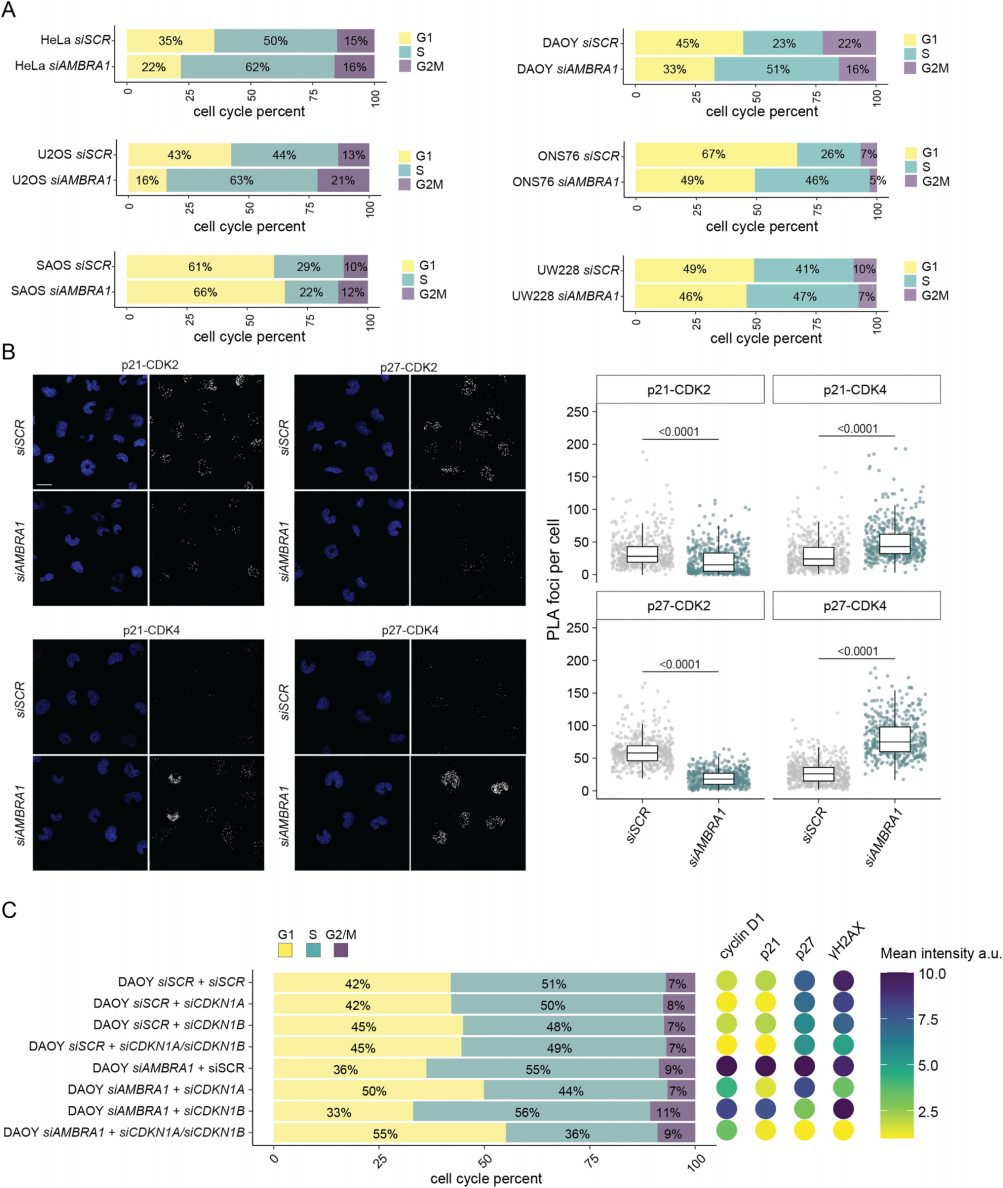
AMBRA1 regulates the G1/S phase transition and DDR via the stabilization of cyclin D1 and p21. (**A**) Stacked barplot of cell cycle distribution (%) of QIBC analysis for a scramble- or AMBRA1-depleted HeLa (Top Left), U2OS (Middle Left), and SAOS (Bottom Left) stained for EdU and Hoechst (at least 2000 cells were acquired per condition). Stacked barplot of cell cycle distribution (%) of FACS analysis for scramble- or AMBRA1-depleted DAOY (Top Right), ONS76 (Middle Right), and UW228 (Bottom Right), expressing FUCCI-CA reporter (at least 10 000 cells were analyzed per condition), *n* = 3. (**B**) Left: confocal imaging of PLA for the indicated proteins in control or AMBRA1-depleted DAOY cells. Right: boxplot (median values with IQR) of foci number per cell, for each separate PLA reaction (*n* = 1000 from five independent biological replicates). Scale bar 50 µm. (**C**) Stacked bar plot of QIBC analysis for normalized cell cycle phases distribution (%) in DAOY cells depleted for the indicated genes with siRNA, with the respective bubble plot showing normalized mean intensity (a.u.) of the indicated proteins (cyclin D1: *n* = 1000; p21: *n* = 1000; p27: *n* = 1000; γH2AX: *n* = 1000 in three independent technical replicates). To separate the different cell cycle phases, cells were stained for EdU and Hoechst. Unless otherwise stated, data are presented as mean values, and *n* refers to technical independent samples. Data were analyzed using a unpaired Student *t*-test (B).

To further explore the role of AMBRA1 in regulating cyclin D1–p27/p21 and the G1/S transition, we depleted AMBRA1 in several cell types, including the MB cell models, and quantified the proportion of cells at different cell cycle phases (Fig. 4A and Supplementary Fig. S4A). To incorporate the analysis of the DDR dynamics, we utilized the FUCCI system [19], which employs fluorescently tagged Cdt1 and Geminin degrons to distinguish G1, S, and G2/M phases, and quantified γH2AX accumulation across distinct cell cycle stages. Consistent with previous findings, the absence of AMBRA1 resulted in the accumulation of cells in S phase with concomitant DNA damage, but only in the Rb-proficient genetic background [37] (Supplementary Fig. S4A and B). These results support the notion that in the absence of AMBRA1, cells accumulate RS [1].

To validate the role of Rb and the accumulation of S-phase cells in the RS phenotype observed in AMBRA1-deficient cells, we co-depleted AMBRA1 and Rb in ONS76 cells. QIBC analysis demonstrated that Rb depletion alone increased the number of cells in S phase and was associated with an increase in γH2AX signal (Supplementary Fig. S4C). Although co-silencing of AMBRA1 and Rb did not further increase the number of S-phase cells, AMBRA1 depletion further enhanced γH2AX levels. These results suggest that the RS induced by AMBRA1 depletion arises from at least one other source besides accelerated entry into S phase.

To quantitatively evaluate the molecular dynamics occurring among cyclin–CDK in the absence of AMBRA1, we performed PLA. We found that hyper-stabilization of CDK4–p21/p27 caused by AMBRA1 depletion prevents Cip/Kip proteins from associating with CDK2 (Fig. 4B). We also used a fluorescent kinase translocation reporter system that monitors CDK4/6 and CDK2 activities in single cells [20], where phosphorylation-dependent changes in reporter localization reflect kinase activity. Upon AMBRA1 depletion, both kinases displayed increased activity under basal and serum-deprived conditions, driving S-phase entry independently of mitogenic stimuli (Supplementary Fig. S4D). Recent insights into CDK4/6–CDK2 dynamics suggested that the CDK4/6 inhibitor palbociclib inhibits only cyclin D–CDK4/6 dimers, but not trimeric complexes of cyclin D–CDK4/6 with p27. In the first case, palbociclib may prevent the formation of active CDK4-containing complexes (by binding to CDK4) and indirectly block CDK2 by liberating Cip/Kip inhibitors [38]. Additionally, it was found that the FDA-approved CDK4/6 inhibitors have different target spectra, with palbociclib primarily inhibiting CDK4/6, while abemaciclib also targeting CDK2 and CDK1 [39]. Based on this, we hypothesized that, despite being palbociclib-resistant [30], AMBRA1-depleted cells would retain sensitivity to abemaciclib. Indeed, we confirmed our hypothesis using QIBC analysis, which demonstrated that abemaciclib, unlike palbociclib, was able to fully rescue the S-phase shift and maintain cells in G1 by inhibiting both CDK4/6 and CDK2 activity in AMBRA1-deficient cells (Supplementary Fig. S4E–G).

To assess whether AMBRA1 loss leads to compartment-specific redistribution of p21 and p27, we performed QIBC-based nuclear/cytoplasmic segmentation in MB-SHH cell lines using the Cellpose pre-trained model [40]. Nuclear levels of both proteins were markedly upregulated upon AMBRA1 depletion (Supplementary Fig. S4H). A modest but statistically significant upregulation was also detected in the cytoplasmic fraction, indicating that AMBRA1-mediated degradation impacts p21/p27 levels across subcellular compartments and may contribute to non-canonical cytoplasmic functions of these proteins [34, 41].

To gain insight into the effect of hyper-stabilization of p27 and p21 in the CDK4–cyclin D1–p21/p27 holoenzyme, we depleted p21 and p27 individually or in combination with AMBRA1 in DAOY and U2OS cells, and measured the cell-cycle distribution, alongside the protein expression of cyclin D1, p21, p27, and γH2AX by QIBC. Intriguingly, the cell-cycle shift and the DNA damage caused by AMBRA1 depletion could be rescued by depletion of p21 but not of p27, suggesting an epistatic role of p21 (but not p27) in licensing premature entry into, and progression through, S phase (Fig. 4C and Supplementary Fig. S4I). Together, these results indicate that the RS resulting from AMBRA1 depletion largely reflects the altered abundance and stoichiometry of the cyclin D(CDK4/6)–p21 complex, with direct negative consequences for cell cycle homeostasis.

### AMBRA1 regulates replication fork speed and lagging-strand synthesis via p21–PCNA dynamics

**Figure 5.**
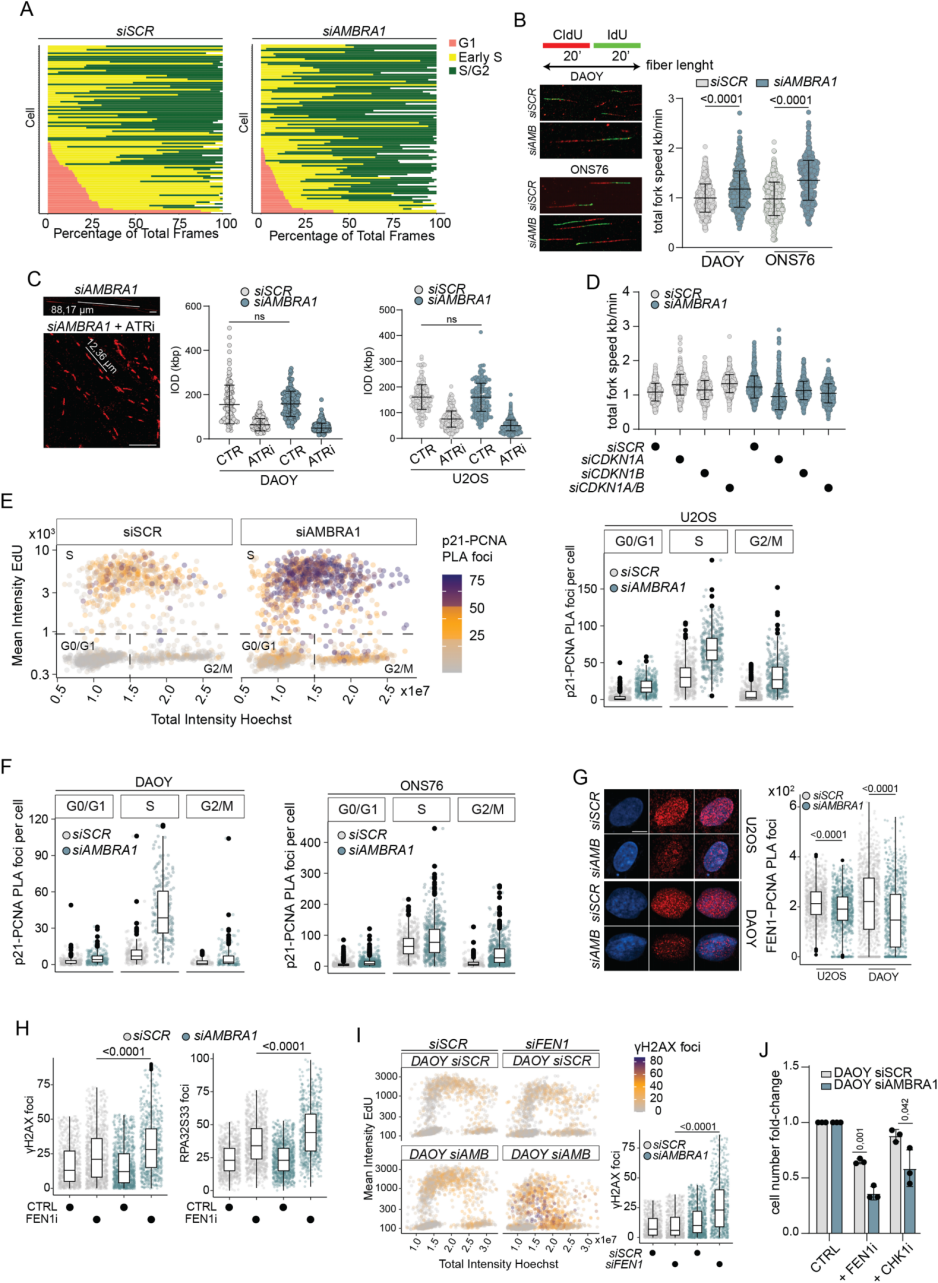
AMBRA1 maintains replication fidelity by modulating p21–PCNA interactions and lagging-strand maturation. (**A**) Live-imaging cell-cycle profiles of individual U2OS-FUCCI cells (each bar = one cell) 24 h post-AMBRA1 silencing, imaged for 48 h (*n* = 100 randomly selected). (**B**) Replication fork speed in AMBRA1-depleted or control DAOY and ONS76 cells (DAOY siSCR: *n* = 546; siAMBRA1: *n* = 537/ONS76 siSCR: *n* = 576; siAMBRA1: *n* = 552). (**C**) Left: representative IOD images. Scatterplot quantification in DAOY (middle) and U2OS (right) ± AMBRA1 depletion ± ATRi (30 min) (DAOY: siSCR CTRL *n* = 126, +ATRi *n* = 229; siAMBRA1 CTRL *n* = 148, +ATRi *n* = 368/U2OS: siSCR CTRL *n* = 162, +ATRi *n* = 296; siAMBRA1 CTRL *n* = 177, +ATRi *n* = 398). (**D**) Replication fork length in U2OS cells treated as in Fig. 4C (siSCR + siSCR: *n* = 513; +siCDKN1A: *n* = 561; +siCDKN1B: *n* = 516; +siCDKN1A/B: *n* = 564/siAMBRA1 + siSCR: *n* = 533; +siCDKN1A: *n* = 527; +siCDKN1B: *n* = 540; +siCDKN1A/B: *n* = 564). Tukey’s post-hoc *P*-values: siSCR + siSCR versus siSCR + siCDKN1A < 0.0001; versus siCDKN1B < 0.0217; versus siAMBRA1 + siSCR < 0.0001; versus siAMBRA1 + siCDKN1A < 0.0001; versus siAMBRA1 + siCDKN1B ns; siAMBRA1 + siSCR versus siAMBRA1 + siCDKN1A < 0.0001; versus siCDKN1B < 0.0001; versus siCDKN1A/B < 0.0001. (**E**) Left: QIBC of p21–PCNA PLA in pre-extracted, EdU/Hoechst-stained U2OS ± AMBRA1 depletion (2000 cells/condition). Right: boxplot quantification (*n* = 400/cell cycle phase). (**F**) p21–PCNA PLA boxplots in pre-extracted DAOY (left) and ONS76 (right) (DAOY: siSCR/siAMBRA1 G1 = 400, S = 400, G2/M = 110/118; ONS76: G1 = 400, S = 400, G2/M = 227/537). (**G**) Representative QIBC images and boxplot of PCNA–FEN1 PLA in U2OS and DAOY ± AMBRA1 depletion (*n* = 1000/condition). Scale bar 10 µm. (**H**) Boxplot of γH2AX (left) and phospho-RPA32(S33) (right) foci in DAOY S-phase cells ± AMBRA1 depletion ± FEN1i (*n* = 1000/condition). (**I**) Left: QIBC of γH2AX foci across the cell cycle in DAOY cells with indicated depletions (2000 cells/condition). Right: boxplot (*n* = 1000/condition). (**J**) Fold-change cell number in DAOY ± AMBRA1 depletion after 48 h treatment with indicated compounds (*n* = 3). Scatter plots: mean ± SD; boxplots: median and IQR. Unless otherwise stated data are presented as mean value ± SD from three biologically independent samples. (A, C–I) One-way ANOVA; (J) unpaired Student’s *t*-test.

We previously demonstrated that AMBRA1 loss promotes genomic instability through the accumulation of RS [1]; however, the precise underlying mechanism remained unclear. Notably, AMBRA1 silencing profoundly altered cell-cycle dynamics, as revealed by live-cell imaging using the FUCCI system. While S-phase entry and its initial steps were accelerated, the late S phase and transition into G2 were prolonged, suggesting the accumulation of RS and a delay in the traversal of cells into G2. Despite this delay, the overall duration of the cell cycle remained shortened in AMBRA1-silenced cells (Fig. 5A). As previously observed in other cell types [1], in the absence of AMBRA1, MB cell lines showed an accelerated speed of replication fork progression, accompanied by the accumulation of asymmetric forks (Fig. 5B and Supplementary Fig. S5A). Fast replication fork speed may result from reduced origin firing. When fewer replication origins are activated, existing replication forks compensate by increasing their speed to ensure timely completion of genomic DNA replication [42]. To determine whether the effects observed upon AMBRA1 depletion were due to a direct impact on the origin firing or an indirect consequence, we measured inter-origin distance (IOD) both under basal conditions and following ATR inhibitor (ATRi) treatment, which promotes the activation of dormant origins [43, 44]. The results showed no differences in IOD between AMBRA1-depleted and control cells (Fig. 5C). To further investigate the origin of replication activity, we measured chromatin-bound levels of key origin licensing and activation proteins, namely MCM2, CDC6, CDC45, and CDT1, in both MB and non-MB cell lines (Supplementary Fig. S5D–G), and the phospho-MCM2, a surrogate marker of origin activation [45] (Supplementary Fig. S5H). Indeed, no consistent differences were observed in AMBRA1-depleted cells across the three cell lines, suggesting that the RS induced by AMBRA1 deficiency may occur independently of origin activation.

Given the potential critical role of p21 in regulating replication fork progression, and to further substantiate its causal involvement in the AMBRA1-associated phenotype, we assessed replication fork speed following individual or combined depletion of p21, p27, and AMBRA1. Consistent with our previous observation, p21 depletion accelerated fork speed [46], in contrast to p27. Notably, by knocking down p21 in AMBRA1-depleted cells, the speed of fork progression was reduced (Fig. 5D and Supplementary Fig. S5I).

It is known that among all known PIP-box motif-containing proteins, p21 has the highest known affinity for PCNA [47]. Therefore, we tested whether this interaction is altered in the absence of AMBRA1 using a high-content Airyscan microscopy method to quantify the signal of PLA foci in chromatin-bound p21–PCNA complex. The results showed that upon AMBRA1 silencing, the p21–PCNA interaction is significantly enhanced in distinct cell-cycle phases (Fig. 5E and F). The analysis of the chromatin-bound fraction revealed that the enrichment was prominent throughout the entire S phase, suggesting a potential impact on PCNA regulation. This effect is likely driven by a general accumulation of p21 across all phases of the cell cycle in the absence of AMBRA1 (Supplementary Fig. S5K), which may exacerbate the disruption of replication-associated protein interactions.

FEN1, a 5′–3′ exonuclease essential for Okazaki fragment maturation, cannot simultaneously interact with PCNA and p21 [48]. Moreover, p21 overexpression disrupts the FEN1–PCNA interaction, and reduced FEN1 levels are associated with accelerated replication fork progression [46]. Therefore, we hypothesized that the hyper-stabilization of p21 might impair lagging-strand synthesis by altering the FEN1–PCNA interaction. To test this, high-content PLA analysis of nuclear FEN1–PCNA interactions was performed, revealing a significant decrease in their association upon AMBRA1 depletion (Fig. 5G), despite the fact that more cells are in S phase [1]. Next, we tested whether the acceleration of forks together with the altered processivity of the PCNA–p21/FEN1 complex could leave under-replicated DNA, and performed the S1-nuclease assay to reveal single-stranded DNA gaps [49]. Indeed, in the absence of AMBRA1, DNA replication forks were sensitive to the S1 digestion (Supplementary Fig. S6A). In parallel, QIBC analysis revealed that further compromising lagging-strand synthesis, either by pharmacological inhibition or siRNA-mediated silencing of FEN1, exacerbated the extent of DNA damage in AMBRA1-depleted cells. This was reflected by higher γH2AX levels, enhanced RPA32 (S33) foci formation, and alterations in cell-cycle distribution (Fig. 5H and I). To assess the functional consequences of these effects, we performed cell viability assays in AMBRA1-deficient cells. As a positive control, we included the CHK1/2 inhibitor AZD7762, which we previously reported to induce synthetic lethality when combined with AMBRA1 depletion [1]. Accordingly, low AMBRA1 expression sensitized DAOY cells to FEN1 or CHK1 inhibitors, whereas no effect was observed in the ONS76 cells, which already exhibited a robust decrease in cell viability upon AMBRA1 depletion alone (Fig. 5J and Supplementary Fig. S6B and C).

These results collectively highlight the essential role of AMBRA1 in ensuring proper DNA replication by regulating p21 dynamics at the replisome.

### *TP53* status dictates sensitivity to AMBRA1 depletion and RS in MB-SHH

**Figure 6.**
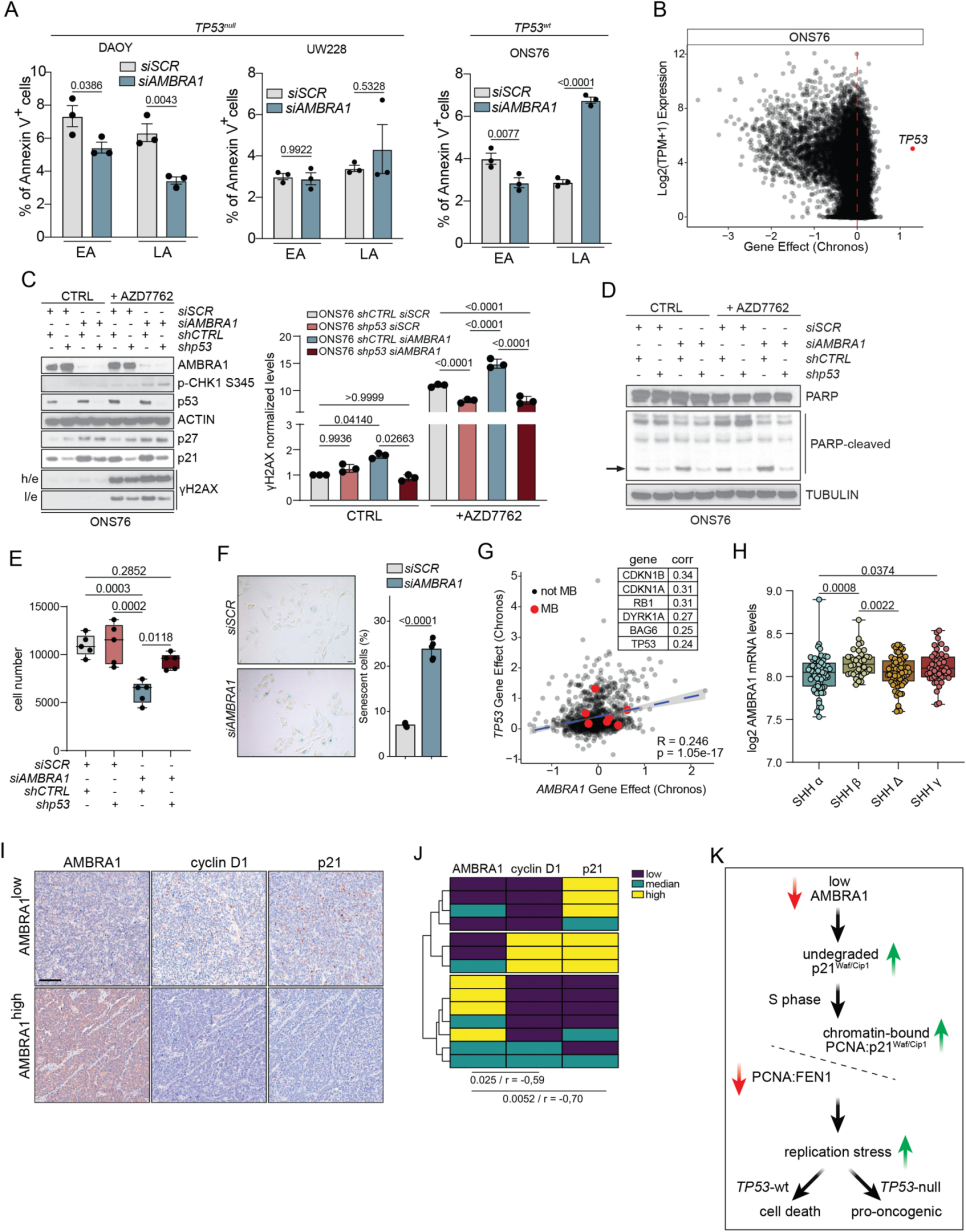
*AMBRA1–TP53* dependency links RS response to cell viability in MB. (**A**) Barplot of Annexin V^+^ cells (%) in early and late apoptotic phase (EA–LA) detected by flow cytometry with Annexin V-APC staining and PI counterstain, in DAOY, UW228, and ONS76 in control or AMBRA1-depleted condition (*n* = 3). (**B**) Scatter plot distribution of Chronos corrected GeneEffect of CRISPR KO over mRNA expression in ONS76 cell line from DepMap. (**C**) Left: IB for the indicated proteins of whole-cell extracts from control (*shCTRL*) or p53 constitutively downregulated ONS76 cells in scramble- or AMBRA1-depleted (*shp53*). Twenty-four hours before harvesting, cells were treated without or with 200 nM AZD7762 (*n* = 3). Right: barplot of γH2AX normalized protein levels (*n* = 3). (**D**) IB for the indicated proteins of whole-cell extracts from control (*shCTRL*) or p53 constitutively downregulated ONS76 cells in scramble or AMBRA1-depleted. Twenty-four hours before harvesting cells were treated with or without 200 nM AZD7762 (*n* = 3). (**E**) Boxplot of cell number from *shCTRL* or *shp53* ONS76 cells in a scramble- or AMBRA1-depleted condition. (**F**) Left: representative brightfield images of SA-β-gal staining in scramble- or AMBRA1-depleted ONS76 cells. Scale bar 25 µm. Right: barplot of the percentage of SA-β-gal-positive cells per condition (*n* = 5 independent biological replicates). (**G**) Left: scatter plot distribution of Chronos corrected GeneEffect of CRISPR KO for *AMBRA1* and *TP53* from 24Q4 DepMap release; MB cell lines in red. Right: table with Top6 co-dependent gene with AMBRA1. (**H**) Boxplot for mRNA log_2_ expression of AMBRA1 in the different SHH subgroups derived from the publicly available dataset Cavalli (763 patients, fpkm normalized, mb500rs1 chip). SHH-α = 65; SHH-β = 35; SHH-Δ = 76; SHH- γ = 47. (**I**) Representative images of IHC for the indicated proteins in our cohort of MB-SHH patients. (**J**) Heatmap of IHC staining levels for the indicated proteins. Scale bar 100 µm. The intensities were ranked in three grades, shown as negative or weak positive (low), median positive (median), and strong positive (high) (*n* = 14). (**K**) A comprehensive model illustrating how reduced AMBRA1 levels lead to the accumulation of RS. Unless otherwise stated data are presented as mean value ± SD. Data were analyzed using two-way ANOVA (A), one-way ANOVA (C and E), unpaired Student’s *t*-test (F), Pearson correlation (G), Welch’s two-tailed unpaired *t*-test (H).

While all three MB cell lines exhibited a similar pattern of RS induction, only the ONS76 displayed particularly pronounced DNA damage, which led to an increase in the apoptotic fraction (Fig. 6A), suggesting that these cells are unable to cope with high levels of RS. Notably, the observed molecular and phenotypic differences among these cell lines may stem from variations in their mutational profiles. Interestingly, while ONS76 retains a functional *TP53* (as reported in *cellosaurus.org*), both DAOY and UW228 harbor point mutations in the tumor suppressor (DAOY: G725T/UW228: C464A) [50]. Consistently, DepMap GeneEffect analysis revealed that TP53 KO had the most significant positive effect on cell viability in the ONS76 cells (Fig. 6B). To determine whether the detrimental impact of AMBRA1 silencing on ONS76 cells depends on p53 activity, we engineered these cells for stable p53 downregulation, replicating the conditions observed in the other two MB cell lines. Importantly, IB analysis showed that p53 knockdown could indeed mitigate the γH2AX levels as well as the PARP cleavage caused by AMBRA1 depletion. These results revealed that p53 loss attenuated the γH2AX accumulation and PARP cleavage induced by AMBRA1 depletion, both under basal conditions and following CHK1 inhibition (Fig. 6C and D). Accordingly, the absence of p53 rescued the sharp decline in cell viability, restoring it to control levels (Fig. 6E). Furthermore, given the sustained increase of p21 in ONS76 cells, we assessed whether senescence might contribute alongside apoptosis to the growth-suppressive phenotype. SA-β-gal staining confirmed senescence increase after AMBRA1 depletion (Fig. 6F), suggesting that the TP53-proficient background of the ONS76 cells enables both apoptotic and senescent responses to RS. This genetic interplay between *AMBRA1* and *TP53* is also supported by DepMap GeneEffect analysis, which revealed a strong co-dependency between the two genes, with MB cell lines predominantly aligning along the regression line and *TP53* emerging as the sixth most co-dependent gene with AMBRA1 (Fig. 6G). Reinforcing this relationship at the subtype level, AMBRA1 mRNA levels are lowest in the SHH-α subtype, which is characterized by the highest prevalence of TP53 mutations [51] (Fig. 6H).

Of clinical relevance, immunohistochemical analysis of a cohort of 14 MB-SHH patient samples revealed an inverse correlation between AMBRA1 and both cyclin D1 and p21 levels. This observation highlights a potential role for AMBRA1 in controlling the stability of these proteins in the SHH subgroup *in vivo*, where deregulation of cell-cycle regulators is a key driver of tumor progression. Importantly, tumors with high AMBRA1 expression exhibited reduced levels of cyclin D1 and p21, further supporting AMBRA1’s function as a regulator of their degradation in this clinically relevant context (Fig. 6I and J).

## Discussion

Despite decades of intense research on Cip/Kip protein families, p21 and p27 are still often considered mere cell cycle inhibitors [52–55]; however, multiple lines of evidence, including the present manuscript, expand the perception of these two key regulatory components of the CDK4/6 kinase complex [56]. In particular, our data provide insight into the coordinated degradation pathways of p21, p27, and D-type cyclins, highlighting a key role for the substrate receptor AMBRA1 in mediating their turnover via the CRL4^DDB1^ complex. This finely tuned regulation occurs throughout the cell cycle and is critical for maintaining the appropriate stoichiometry and function of the CDK4/6–cyclin D–p21/p27 complex, which in turn supports proper S-phase entry and safeguards DNA replication fidelity. Importantly, our findings add to a growing appreciation of the nuanced, context-dependent interplay between p21, p27, Cyclin D1, and their associated complexes. These proteins can mutually regulate each other’s stability and function, resulting in complex network dynamics that are sensitive to cell type, signaling cues, and genetic background. Our results reinforce the notion that dysregulation of any single component can propagate through the network to influence cell-cycle progression and cellular responses to RS.

An important open question concerns the molecular basis of AMBRA1 substrate recognition for p21 and p27. Recent structural and biochemical work identified a phospho-degron in the C-terminal region of cyclin D as the critical determinant for CRL4^AMBRA1^ substrate engagement [57]. By analogy, it is tempting to speculate that AMBRA1 recognizes p21 and p27 through phosphorylation-dependent degron motifs. Both proteins are subject to extensive phosphorylation-mediated regulation: p27 is phosphorylated at multiple sites (T157, T187, and S10) [58] that modulate its subcellular localization and stability, while p21 stability is regulated by phosphorylation at T145 and S114, among others [34]. Whether any of these modifications constitute a bona fide phospho-degron recognized by the AMBRA1, analogously to the cyclin D C-terminal motif, remains to be formally established and represents an important avenue for future structural investigation.

Notably, the observation that p21 and p27 accumulation is only partially reduced—rather than fully abrogated—upon cyclin D depletion in AMBRA1-depleted cells suggests that cyclin D contributes to, but does not solely account for, the AMBRA1-mediated regulation of these proteins. This reflects the inherent complexity of a regulatory network in which multiple inputs converge on the same substrates and underscores the importance of considering stoichiometric and competitive relationships within the CDK4/6–cyclin D–p21/p27 holoenzyme when interpreting loss-of-function phenotypes.

The additive effect of combined AMBRA1 and Cdt2 depletion on p21 accumulation further supports the existence of mechanistically distinct, parallel regulatory arms. Beyond operating through different substrate receptors within the CRL4^DDB1^ complex, AMBRA1 and Cdt2 may also act within temporally distinct windows of the cell cycle: the Cdt2-mediated degradation of p21 is primarily triggered by PCNA loading following UV-induced DNA damage and is therefore most active in S phase in the context of genotoxic stress. By contrast, AMBRA1-dependent regulation, as shown here, appears to operate throughout the cell cycle under basal conditions, maintaining constitutive p21 and p27 turnover independently of DNA damage signaling. This partitioning would allow cells to fine-tune p21/p27 levels in a context-dependent manner, with AMBRA1 providing homeostatic control and Cdt2 acting as a damage-responsive switch.

Beyond its well-established role in regulating the G1 checkpoint, p21 also coordinates DNA replication dynamics and influences the cellular response to RS [59, 60]. Indeed, chronic deregulation of p21 levels has been shown to drive genomic instability through its impact on DNA replication [61]. Previously, in an Rb-null model of senescence evasion, we demonstrated that chronic overexpression of p21 negatively impacts the speed of replication forks [62]. Conversely, we also observed the opposite effect upon p21 depletion, where the replication fork speed is enhanced in a PARP1-dependent manner [46]. Here, we enrich the broad spectrum of p21 involvement in replisome processivity by mechanistically defining how its acute stabilization, combined with the accelerated transition from G1 to S phase, caused by AMBRA1 silencing, affects the dynamic interaction between PCNA and its partners. Dysregulated levels of p21 negatively impact the lagging-strand DNA synthesis, enhancing RS and making AMBRA1-depleted cells sensitive to FEN1 inhibitors (Fig. 6K). Our results suggest that p21 levels are tightly regulated during S phase to ensure proper DNA replication. Notably, AMBRA1 depletion disrupts this balance, resulting in the aberrant regulation of p21. Interestingly, both p21 depletion and enhanced protein stabilization/overabundance result in accelerated replication fork progression. This phenomenon is likely attributable to dysregulated synthesis of the lagging DNA strand in both scenarios.

Beyond its well-documented nuclear roles, p21 and p27 are known to exert non-canonical functions in the cytoplasm, including the regulation of apoptosis, cytoskeletal dynamics, and cell migration [34, 41]. QIBC-based subcellular segmentation in MB-SHH cells revealed that AMBRA1 depletion results in pronounced nuclear upregulation of both proteins, alongside a modest yet significant increase in the cytoplasmic fraction, indicating that impact of AMBRA1-mediated degradation is not restricted to the nucleus. While the nuclear accumulation of p21 is directly linked to the RS phenotype described above, the concurrent cytoplasmic elevation may contribute additional facets to the AMBRA1-depleted phenotype, including altered apoptotic threshold or cytoskeletal reorganization, aspects that warrant further investigation.

In our attempt to find a specific tumor context highly reliant on this aberrant modulation, we identified a defined subset of MBs, namely MB-SHH, where we demonstrate the importance of AMBRA1 downregulation in enabling tumor cell proliferation independently of mitogenic signaling. Our results also document the relevance of the G1/S regulatory function of AMBRA1 in cerebellar development, potentially shedding new light on the involvement of this molecular axis in other cerebellar-related disorders. Additionally, by using genetic, biochemical, and pharmacological approaches, we mechanistically define how AMBRA1 depletion concomitantly exacerbates CDK4/6 as well as CDK2 activity, leading to palbociclib resistance in MB-SHH cell lines. Nonetheless, we identify an alternative CDK4/6 inhibitor, abemaciclib, as a promising therapeutic approach, given the significant repressive effect this compound has on CDK2 as well [39].

The observation that AMBRA1 depletion induces both apoptosis and senescence in TP53-proficient ONS76 cells provides a framework to interpret the ONS76 phenotype in the context of p53’s diverse functions. Notably, p53 transcriptionally induces p21, which, in the absence of AMBRA1, is further stabilized post-translationally, amplifying its accumulation beyond what either perturbation would achieve alone. Consistently with the well-established role of p21 as a downstream effector of p53-dependent senescence, our data suggest that this compounded elevation of p21 is associated with the engagement of senescence programs. One could therefore speculate that the apoptotic outcome observed in ONS76 cells upon AMBRA1 loss may reflect at least two cell-fate-determining roles of p53 in this particular cell line: (i) p53-mediated transcriptional induction of p21 (in response to DNA damage), which—when further stabilized by AMBRA1 absence—exacerbates RS, and may promote senescence entry; and (ii) p53’s role as a sensor and transactivator of the DDR, which translates suprathreshold RS-associated DNA damage into the cell’s apoptotic commitment.

Of clinical relevance, a previous observation from a SB transposon-based screening identified AMBRA1 as one of the most frequently mutated genes in a cohort of embryonal tumors of the nervous system primed by p53 mutation [16]. In agreement with that, we describe how p53 mutation supports MB-SHH cells in coping with the intrinsic genomic instability caused by AMBRA1 deficiency, licensing aberrant S phase entry and promoting apoptotic evasion. While our data from both cell lines and tumor samples support a potential role for AMBRA1 in SHH-MB, further studies incorporating clinically relevant models and larger patient cohorts will be essential to conclusively define its contribution to SHH-MB initiation and progression *in vivo*.

In summary, our findings establish AMBRA1 as a critical regulator of S phase entry and DNA replication fidelity through its dual role in controlling p21 turnover and cyclin D expression. Depletion of AMBRA1 leads to aberrant stabilization of the CDK4/6–cyclin D–p21/p27 complex, accelerated G1/S transition, and impaired coordination of lagging strand synthesis, ultimately promoting RS and genomic instability. Our work not only uncovers a mechanistic basis for AMBRA1’s tumor suppressor function in SHH-MB but also highlights abemaciclib and FEN1 inhibition as a potential therapeutic strategy to counteract the therapy resistance conferred by AMBRA1 deficiency. These insights have direct implications for the development of targeted therapies and biomarker-driven patient stratification in MB, as well as potentially for other AMBRA1-deficient cancers.

## Supplementary Material

gkag595_Supplemental_Files

## Data Availability

Kaplan–Meier analysis in Fig. 1B and mRNA levels of SHH-MB patients in Figs 1E and 6G referenced during the study are available in public repositories. The dataset GSE85217 can be accessed through GEO at https://www.ncbi.nlm.nih.gov/geo/query/acc.cgi?acc=GSE85217. The “Pomeroy dataset” is available through the R2 Genomics Analysis and Visualization Platform (https://r2.amc.nl/) under the identifier ps_avgpres_pomeroypublic204_u133a. Access to the platform requires registration for a free user account. Codependencies analysis in Figs 1A and 6B, G are available in the public repository DepMap (https://depmap.org/portal/gene/AMBRA1). Raw proteomic analysis is uploaded in the PRIDE repository with dataset identifier PXD053083. Data of the processed proteomic analysis displayed in Fig. 2A–C can be found in Supplementary Table 1. The data underlying this article will be shared on reasonable request to the corresponding authors.
