# Supplementary material for: Ubiquitin-dependent degradation of p27Kip1 and p21Waf1/Cip1 by AMBRA1 ensures G1 and S phase progression and limits replication stress
Source: Nucleic Acids Res. 2026 Jun 24;54(12):gkag595. doi: 10.1093/nar/gkag595 (PMC13291606; doi:10.1093/nar/gkag595)
Supplement: gkag595_Supplemental_Files [file gkag595_supplemental_files.zip › Supplementary Figures.pdf]

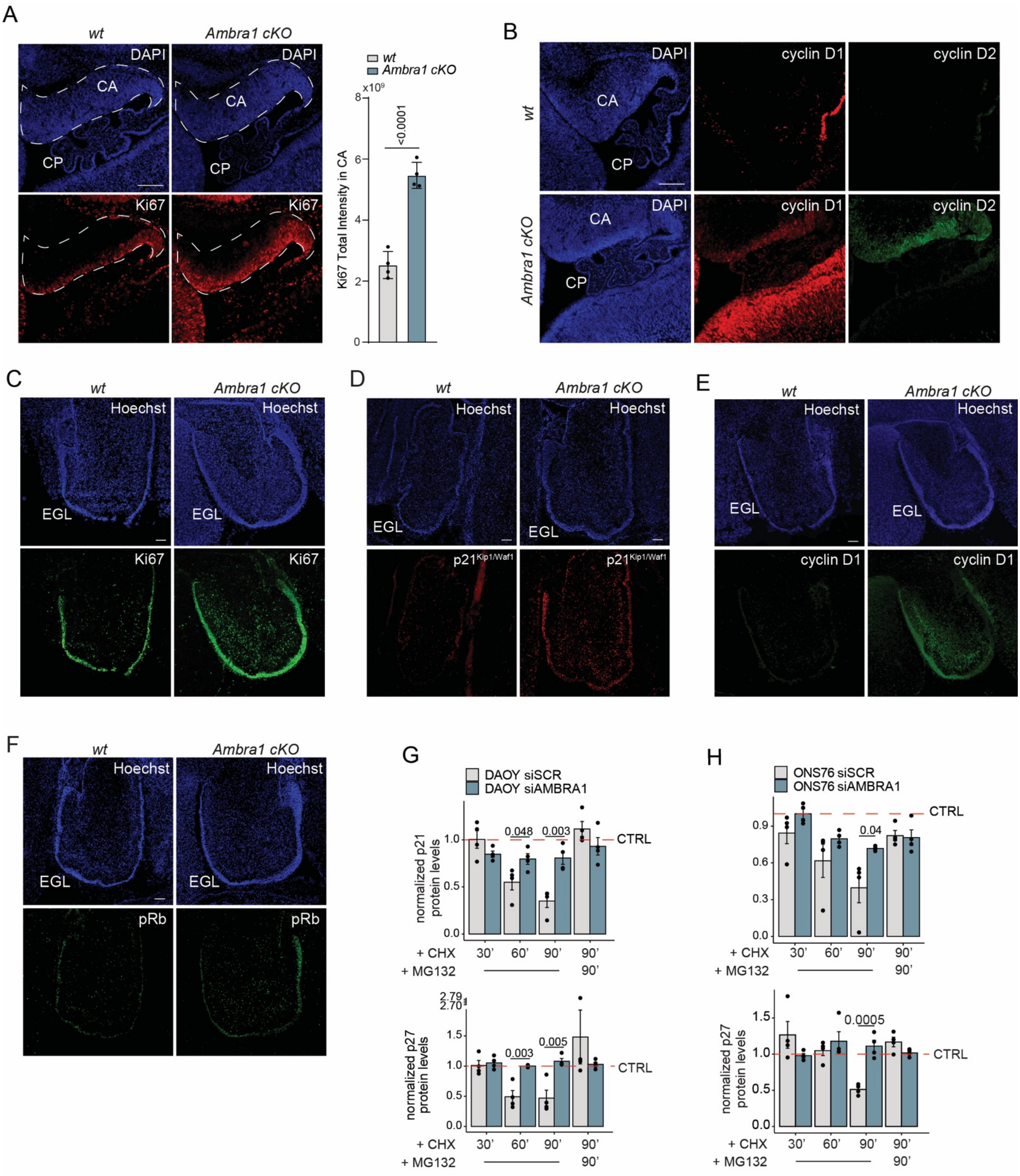

A
wt
Ambra1 cKO
DAPI
CA
CP
Ki67
wt
Ambra1 cKO
x10^9
<0.0001
Ki67 Total Intensity in CA
B
DAPI
cyclin D1
cyclin D2
wt
Ambra1 cKO
C
Hoechst
EGL
Ki67
D
p21Kip1/Waf1
E
cyclin D1
F
pRb
G
DAOY siSCR
DAOY siAMBRA1
normalized p21 protein levels
0.048
0.003
CTRL
+ CHX
+ MG132
30'
60'
90'
normalized p27 protein levels
2.79
2.70
0.003
0.005
H
ONS76 siSCR
ONS76 siAMBRA1
0.04
0.0005

Fig. S1. Ambra1 controls cerebellar development through cell cycle regulation.

(A) Sagittal sections from wild-type and Ambra1 cKO E13.5 embryos (Top), stained for Ki67 and Hoechst (n=4). Dashed area refers to CA, cerebellar anlage; CP, choroid plexus. Right, Barplot of Ki67 total intensity in the CA. **(B)** Sagittal sections from wild-type and Ambra1 cKO E13.5 embryos stained for cyclin D1, cyclin D2 and Hoechst (n=4). **(C to F)** Sagittal sections from wild-type and Ambra1 cKO E18.5 embryos stained for Ki67 **(C)**, p21 **(D)**, cyclin D1 **(E)**, pRb-S807/811 **(F)** and Hoechst (n=4 – Ki67 p21, cyclin D1; n=3 – pRb). Scale bars 100 μm. **(G to H)** Barplot representing over-CTRL normalized quantification of p21 (Top) and p27 (Bottom) protein levels in DAOY **(G)** and ONS76 **(H)** cells in control or AMBRA1-depleted conditions (shown in Fig. 1G). Unless otherwise stated data are presented as mean value ±SEM and n refers to biological independent samples. Data were analyzed using unpaired Student t-test (A, G and H).

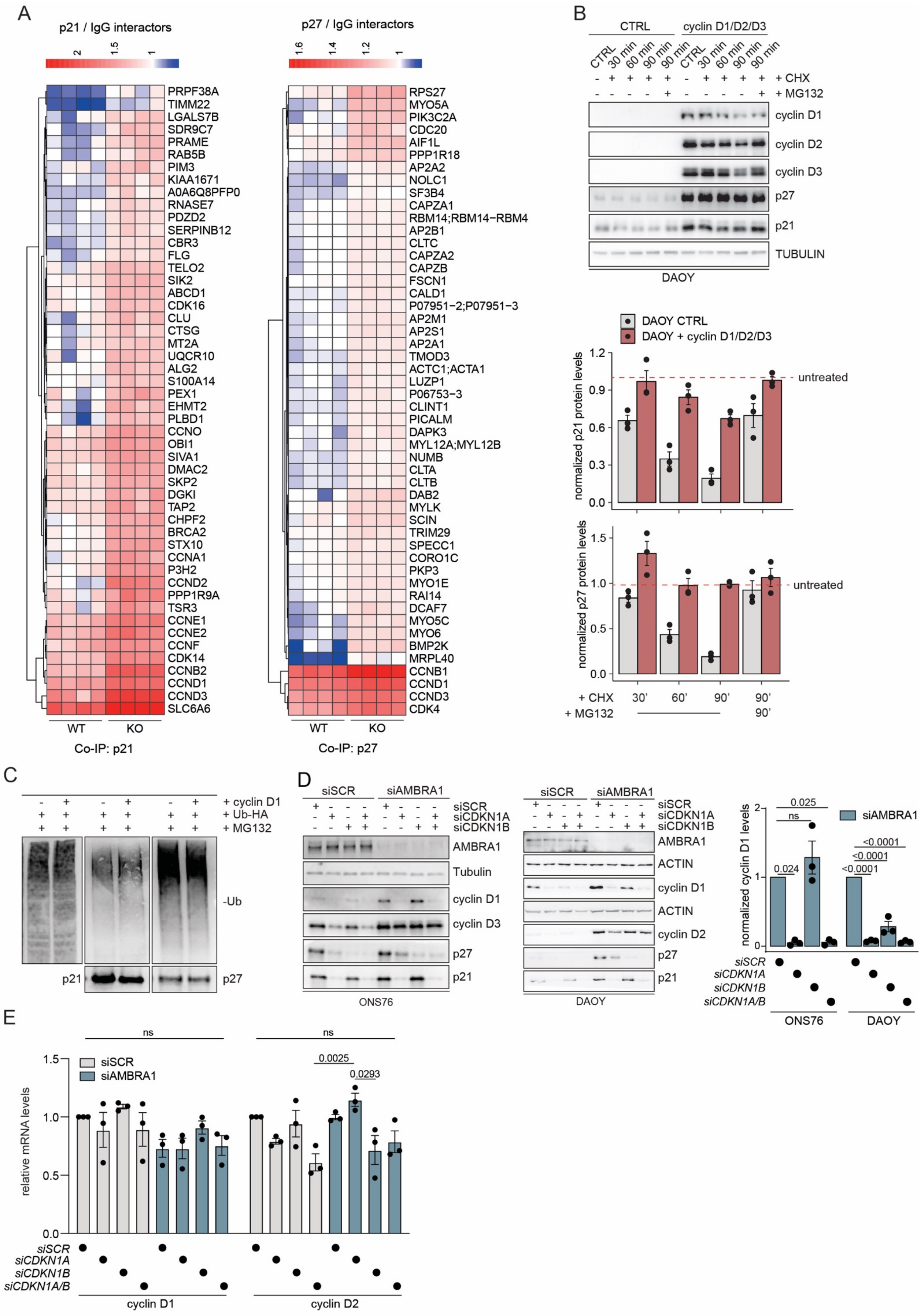

A
p21 / IgG interactors
2
1.5
1
PRPF38A
TIMM22
LGALS7B
SDR9C7
PRAME
RAB5B
PIM3
KIAA1671
A0A6Q8PFP0
RNASE7
PDZD2
SERPINB12
CBR3
FLG
TELO2
SIK2
ABCD1
CDK16
CLU
CTSG
MT2A
UQCR10
ALG2
S100A14
PEX1
EHMT2
PLBD1
CCNO
OBI1
SIVA1
DMAC2
SKP2
DGKI
TAP2
CHPF2
BRCA2
STX10
CCNA1
P3H2
CCND2
PPP1R9A
TSR3
CCNE1
CCNE2
CCNF
CDK14
CCNB2
CCND1
CCND3
SLC6A6
WT
KO
Co-IP: p21
p27 / IgG interactors
1.6
1.4
1.2
1
RPS27
MYO5A
PIK3C2A
CDC20
AIF1L
PPP1R18
AP2A2
NOLC1
SF3B4
CAPZA1
RBM14;RBM14-RBM4
AP2B1
CLTC
CAPZA2
CAPZB
FSCN1
CALD1
P07951-2;P07951-3
AP2M1
AP2S1
AP2A1
TMOD3
ACTC1;ACTA1
LUZP1
P06753-3
CLINT1
PICALM
DAPK3
MYL12A;MYL12B
NUMB
CLTA
CLTB
DAB2
MYLK
SCIN
TRIM29
SPECC1
CORO1C
PKP3
MYO1E
RAI14
DCAF7
MYO5C
MYO6
BMP2K
MRPL40
CCNB1
CCND1
CCND3
CDK4
WT
KO
Co-IP: p27
B
CTRL
cyclin D1/D2/D3
CTRL 30 min 60 min 90 min 90 min CTRL 30 min 60 min 90 min 90 min
+ CHX
+ MG132
cyclin D1
cyclin D2
cyclin D3
p27
p21
TUBULIN
DAOY
DAOY CTRL
DAOY + cyclin D1/D2/D3
normalized p21 protein levels
untreated
normalized p27 protein levels
untreated
+ CHX 30' 60' 90' 90'
+ MG132 90'
C
+ cyclin D1
+ Ub-HA
+ MG132
-Ub
p21
p27
D
siSCR
siAMBRA1
siSCR
siCDKN1A
siCDKN1B
AMBRA1
Tubulin
cyclin D1
cyclin D3
p27
p21
ONS76
AMBRA1
ACTIN
cyclin D1
ACTIN
cyclin D2
p27
p21
DAOY
siAMBRA1
normalized cyclin D1 levels
0.025
ns
0.024
<0.0001
<0.0001
<0.0001
siSCR
siCDKN1A
siCDKN1B
siCDKN1A/B
ONS76
DAOY
E
ns
ns
0.0025
0.0293
siSCR
siAMBRA1
relative mRNA levels
siSCR
siCDKN1A
siCDKN1B
siCDKN1A/B
cyclin D1
cyclin D2

Fig. S2. p21 levels affect cyclins D stability.

(A) Heatmap of top50-upregulated interactions (log2FC) from proteomics analysis in wt and AMBRA1-KO HeLa cells overexpressed with either p21 (Left) or p27 (Right) Flag-tagged plasmids, normalized over the respective IgG control samples (n=4). **(B)** Top: IB of the indicated proteins in DAOY cells overexpressing the three D-type cyclins, treated with 100 µg/ml cycloheximide (CHX) and/or 10 µM MG132 for the indicated time points. Corresponding barplots representing over-untreated normalized quantification of p21 (Middle) and p27 (Bottom) protein levels (n=3). **(C)** IB for the in vivo ubiquitination levels of p21 and p27. Hela cells constitutively overexpressing a doxycycline-inducible form of cyclin D1 were transfected to overexpress a plasmid encoding for an HA-tagged form of ubiquitin and treated with 5 µM MG132 before harvesting as indicated (n=3). **(D)** IB for the indicated proteins in ONS76 (Left) and DAOY (Middle) cells silenced or not for the indicated genes (n=3). Right: Corresponding barplot of siSCR-normalized protein levels of cyclin D1 in AMBRA1-silenced cells. **(E)** Barplot of normalized mRNA levels of CCND1 and CCND2 by q-PCR in DAOY treated as in c (n=3). Unless otherwise stated data are presented as mean value ±SEM and n refers to biological independent samples. Data were analyzed using unpaired Student t-test (B) or One-way ANOVA (D and E).

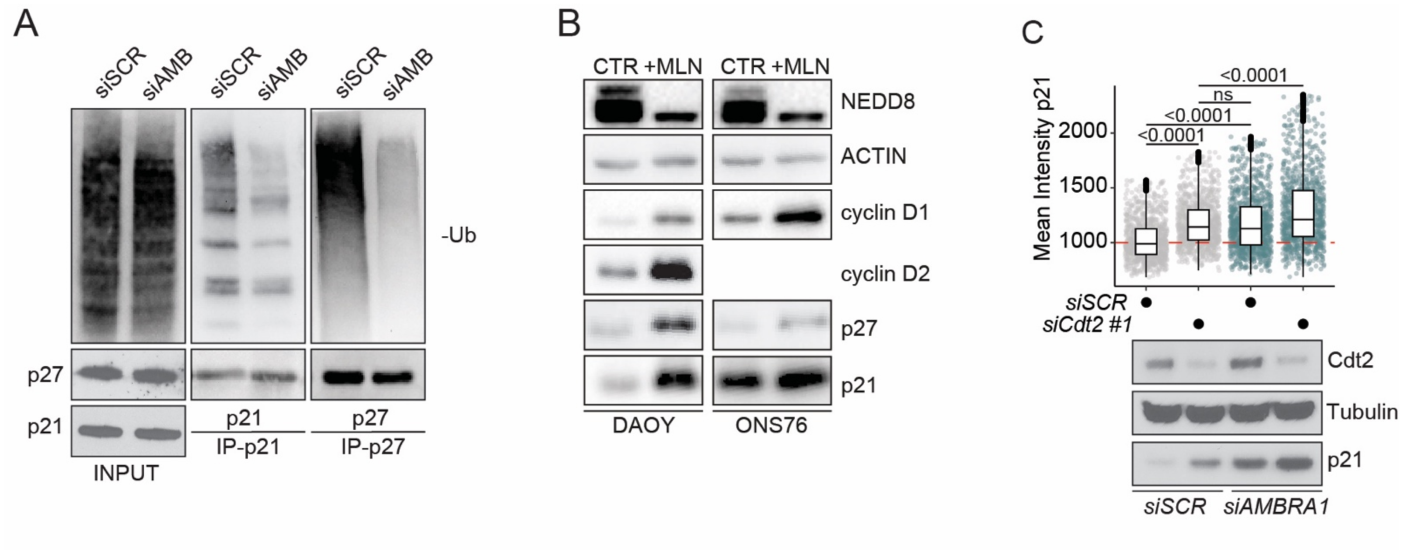

Fig. S3. AMBRA1 depletion impairs DDB1-mediated degradation of p21 and p27.

(A) IB for the in vivo ubiquitination levels of p21 and p27. HA-tagged ubiquitin-overexpressing DAOY cells were silenced or not for AMBRA1 and treated with 5 μM MG132 before harvesting (n=3). **(B)** IB of the indicate proteins in in DAOY and ONS76 cells treated or not with NEDD8 inhibitor 2,5 μM MLN4924 for 4 hours (hrs) (n=3). **(C)** Top: boxplot for QIBC (median values with IQR) analysis for the indicated proteins in DAOY cells silenced or not for the indicated genes (n=1000 in 3 independent technical replicates). Bottom: IB of cells treated as previously indicated (n=2). Unless otherwise stated n refers to biological independent samples. (C) Data were analyzed using One-way ANOVA.

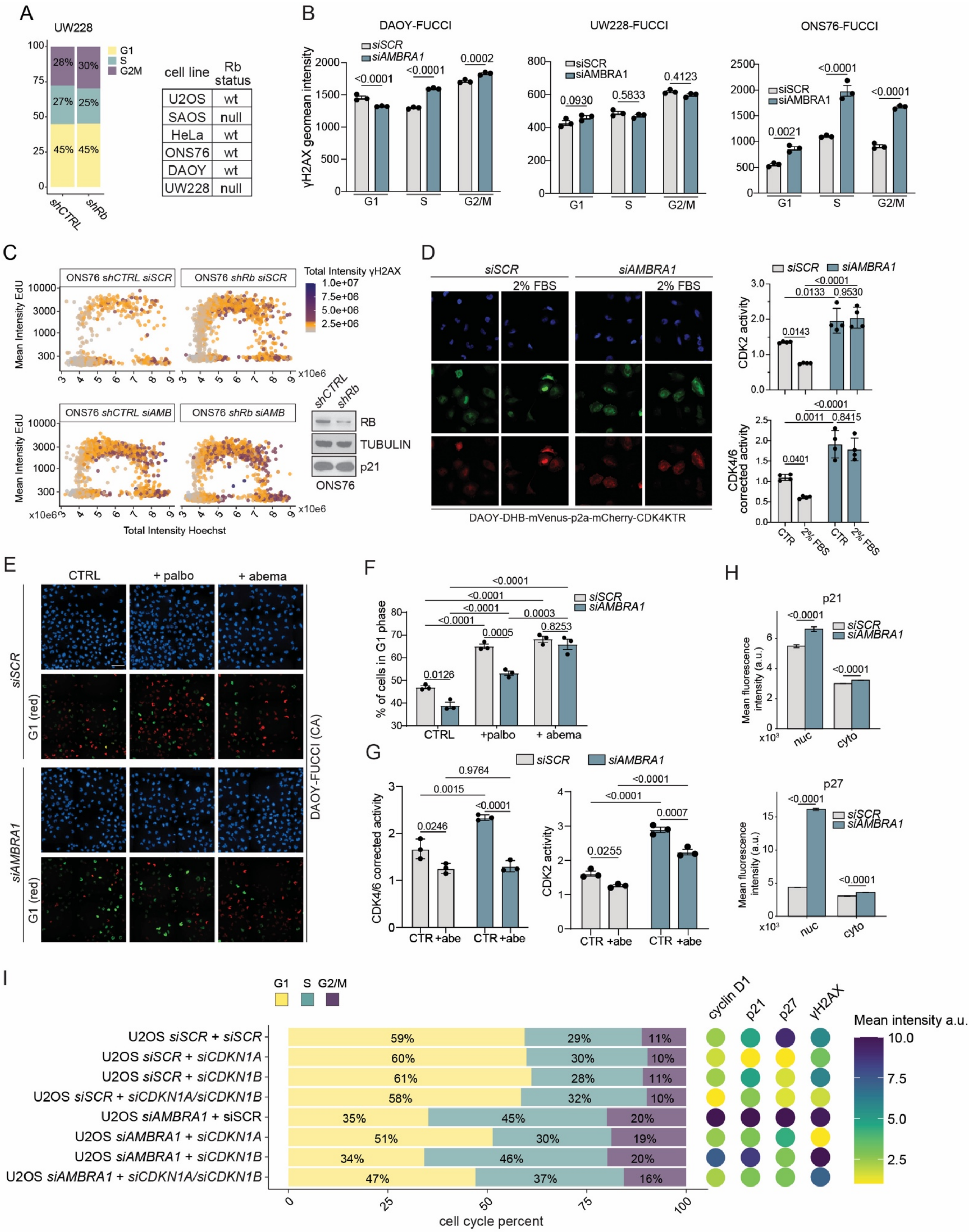

A
UW228
G1
S
G2M
28%
30%
27%
25%
45%
45%
shCTRL
shRb
cell line
Rb status
U2OS wt
SAOS null
HeLa wt
ONS76 wt
DAOY wt
UW228 null
B
DAOY-FUCCI
UW228-FUCCI
ONS76-FUCCI
γH2AX geomean intensity
siSCR
siAMBRA1
G1
S
G2/M
C
ONS76 shCTRL siSCR
ONS76 shRb siSCR
ONS76 shCTRL siAMB
ONS76 shRb siAMB
Total Intensity γH2AX
Mean Intensity EdU
Total Intensity Hoechst
RB
TUBULIN
p21
ONS76
D
siSCR
siAMBRA1
2% FBS
DAOY-DHB-mVenus-p2a-mCherry-CDK4KTR
CDK2 activity
CDK4/6 corrected activity
CTR
2% FBS
E
CTRL
+ palbo
+ abema
G1 (red)
DAOY-FUCCI (CA)
F
% of cells in G1 phase
CTRL
+palbo
+ abema
G
CDK4/6 corrected activity
CDK2 activity
CTR +abe
H
p21
p27
Mean fluorescence intensity (a.u.)
nuc
cyto
I
U2OS siSCR + siSCR
U2OS siSCR + siCDKN1A
U2OS siSCR + siCDKN1B
U2OS siSCR + siCDKN1A/siCDKN1B
U2OS siAMBRA1 + siSCR
U2OS siAMBRA1 + siCDKN1A
U2OS siAMBRA1 + siCDKN1B
U2OS siAMBRA1 + siCDKN1A/siCDKN1B
cyclin D1
p21
p27
γH2AX
Mean intensity a.u.
cell cycle percent

Fig. S4. DNA damage induction following AMBRA1 depletion is partially independent of altered G1/S transition.

(A) Left: Stacked barplot of UW228 in control condition constitutively downregulated for Rb gene expression (shRb) (n = 400 in 3 biological independent replicates). Right: table summarizing Rb status across the cell lines used in this study. **(B)** Barplot of γH2AX geomean intensity analyzed by flow cytometry in DAOY-FUCCI (CA), UW228-FUCCI (CA) and ONS76-FUCCI (CA) in control or AMBRA1-depleted condition (n=10000 events in three independent technical replicates). **(C)** Left: QIBC analysis of γH2AX total intensity along cell cycle of control (shCTRL) or shRb ONS76 cells in scramble or AMBRA1-depleted condition stained for EdU and Hoechst (2000 cells are displayed per condition). Right: IB for the indicated proteins of whole-cell extracts from of control (shCTRL) or shRb ONS76. **(D)** Left: representative images from DAOY cells engineered to constitutively express a CDK4/6 (red) and CDK2 (green) activity reporter (n=4). Scale bar 100 μm. Right: barplot of CDK2 activity (Top) and CDK4/6 corrected activity (Bottom). Cells were depleted for scramble or AMBRA1 (siRNA) and FBS-deprived (FBS 2%) or not for 24 hrs and then analyzed by high-content imaging. **(E)** Representative images of DAOY-FUCCI (CA) cells treated for 24 hrs with 1μM palbociclib and 500 nM abemaciclib and analyzed by high-content imaging. Scale bar 100 μm. **(F)** Barplot for G1 phase cells % analyzed in (G) (n=3). **(G)** Barplot for CDK4/6 (Left) and CDK2 (Right) activity reporter. Cells were depleted for scramble or AMBRA1 (siRNA) and then treated or not with 500 nM abemaciclib for 24 hrs and then analyzed by high-content imaging (n=3). **(H)** Barplot of QIBC analysis of nuclear (nuc) and cytoplasmic (cyto) mean intensity (a.u.) of p21 (Up) and p27 (Down) in scramble or AMBRA1-depleted DAOY cells, segmented using the Cellpose model (at least 1000 cells per condition per replicate, in three independent biological replicates). **(I)** Stacked bar plot of QIBC analysis for normalized cell cycle phases distribution (%) in U2OS cells treated as in Fig 3C, with the respective bubble plot showing normalized mean intensity (a.u.) of the indicated proteins (cyclin D1: n=1000; p21: n=1000; p27: n= 1000; γH2AX: n=1000 in three independent technical replicates). Unless otherwise stated data are presented as mean value ±SEM and n refers to biological independent samples. [(F), (G)] Data were analyzed using One-way ANOVA, (H) was analyzed by using unpaired Student t-test.

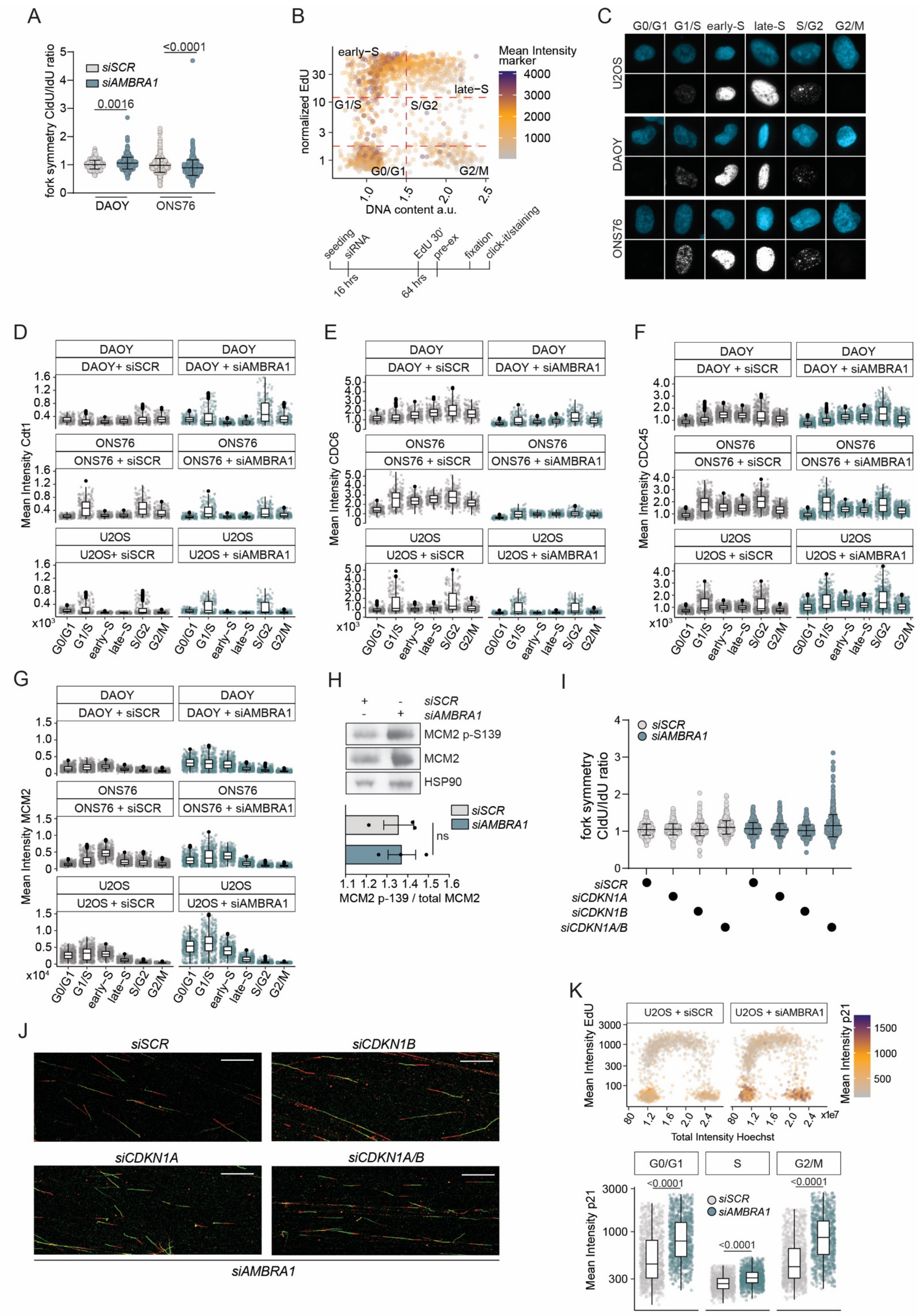

A
fork symmetry CldU/IdU ratio
siSCR
siAMBRA1
0.0016
<0.0001
DAOY
ONS76
B
early−S
late−S
G1/S
S/G2
G0/G1
G2/M
normalized EdU
DNA content a.u.
Mean Intensity marker
4000
3000
2000
1000
seeding
siRNA
16 hrs
EdU 30' pre-ex
64 hrs
fixation
click-it/staining
C
G0/G1
G1/S
early-S
late-S
S/G2
G2/M
U2OS
DAOY
ONS76
D
DAOY
DAOY+ siSCR
DAOY + siAMBRA1
ONS76
ONS76 + siSCR
ONS76 + siAMBRA1
U2OS
U2OS+ siSCR
U2OS + siAMBRA1
Mean Intensity Cdt1
x10³
E
DAOY + siSCR
DAOY + siAMBRA1
ONS76 + siSCR
ONS76 + siAMBRA1
U2OS + siSCR
U2OS + siAMBRA1
Mean Intensity CDC6
F
Mean Intensity CDC45
G
Mean Intensity MCM2
x10⁴
H
siSCR
siAMBRA1
MCM2 p-S139
MCM2
HSP90
ns
MCM2 p-139 / total MCM2
I
fork symmetry CldU/IdU ratio
siSCR
siCDKN1A
siCDKN1B
siCDKN1A/B
J
siSCR
siCDKN1B
siCDKN1A
siCDKN1A/B
siAMBRA1
K
U2OS + siSCR
U2OS + siAMBRA1
Mean Intensity EdU
Total Intensity Hoechst
x1e7
Mean Intensity p21
G0/G1
S
G2/M
<0.0001
<0.0001
<0.0001

Fig. S5. AMBRA1 depletion alters replisome processivity without affecting origin firing.

(A) Replication fork symmetry from DAOY and ONS76 cells depleted or not for AMBRA1 (DAOY siSCR: n = 546; DAOY siAMBRA1: n = 537 / ONS76 siSCR: n = 576; ONS76 siAMBRA1: n = 552). **(B)** Top: following image acquisition, nuclei or cells are segmented using a customized pipeline based on the Cellpose algorithm. Segmented single-cell data are then visualized in scatter plots according to DNA content and EdU incorporation levels. Bottom: timeline of a representative experimental workflow utilizing Quantitative Image-Based Cytometry (QIBC) technology. **(C)** Representative fluorescence images of U2OS and MB-SHH cells nuclei stained with DAPI (light blue) and EdU (white), illustrating nuclear morphology and DNA replication activity with the corresponding cell cycle phase. **(D to G)** QIBC analysis of chromatin-bound Cdt1 **(D)**, CDC6 **(E)**, CDC45 **(F)** and MCM2 **(G)** in U2OS, DAOY and ONS76 cells, stained for EdU and Hoechst (CDC6, CDC45, MCM2: 500 cells are displayed per cell cycle phase, Cdt1: 200 cells are displayed per cell cycle). **(H)** Top: IB for the indicated proteins of whole-cell extracts in ONS76 cells silenced or not for the indicated genes. Bottom: barplot of corresponding quantification of normalized phosphorylated levels over total (n=3). **(I and J)** Scatterplot analysis of fork symmetry in U2OS cells treated as in Fig. 4C with their corresponding representative images **(J)** (U2OS siSCR + siSCR: n = 513; U2OS siSCR + siCDKN1A: n = 561; U2OS siSCR + siCDKN1B: n = 516; U2OS siSCR + siCDKN1A/B: n = 564; U2OS siAMBRA1 + siSCR: n =533; U2OS siAMBRA1 + siCDKN1A: n = 527; U2OS siAMBRA1 + siCDKN1B: n = 540; U2OS siAMBRA1 + siCDKN1A/B: n = 564; in three technical independent experiments). **(K)** QIBC analysis of control and AMBRA1-depleted U2OS cells stained for p21, EdU and Hoechst. Top: scatter plot for cell cycle distribution and p21 nuclear intensity (2000 cells are displayed per the condition of three independent replicates). Bottom: boxplot (median and IQR) of p21 nuclear intensity (n = 1000 for all cell cycle phases). Data are presented as mean value ±SD for scatter plots while median and IQR are shown for boxplots. n refers to biological independent samples. [(A) and (K)] Data were analyzed using One-way ANOVA. (H) Data were analyzed using unpaired Student t-test.

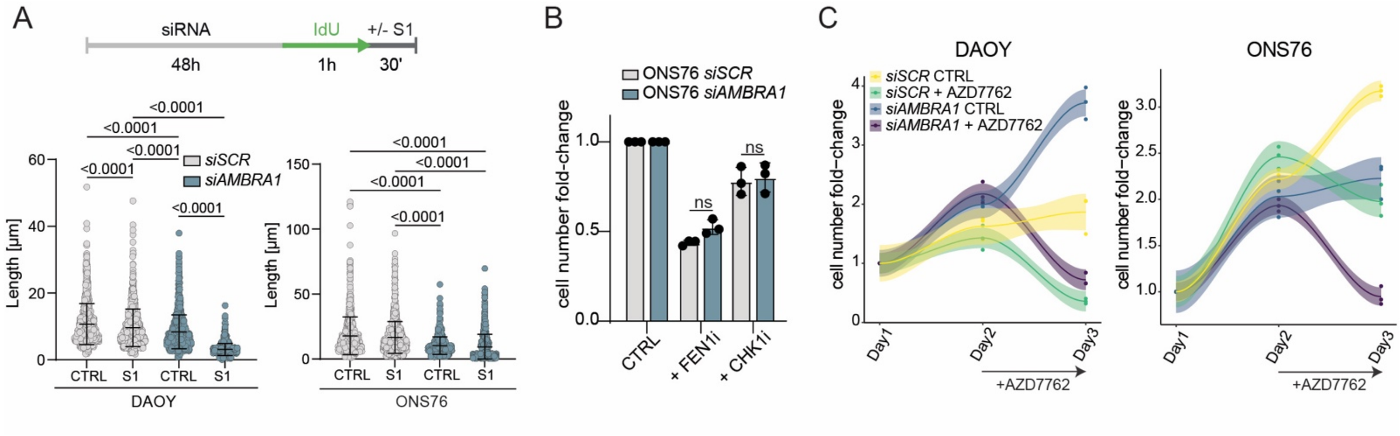

Fig. S6. AMBRA1 depletion sensitizes to lagging strand synthesis inhibition.

(A) Replication fork length in DAOY and ONS76 cells depleted or not for AMBRA1 and treated without (CTRL) or with S1 nuclease (S1) for 30' (DAOY siSCR CTRL: n = 1002; DAOY siSCR + S1: n = 1008; DAOY siAMBRA1 CTRL: n = 1002; DAOY siAMBRA1 + S1: n = 1005 / ONS76 siSCR CTRL: n =1010; ONS76 siSCR + S1: n = 623; ONS76 siAMBRA1 CTRL: n = 764; ONS76 siAMBRA1 + S1: n = 1010; in three technical independent experiments). **(B)** Barplot representing cell number fold-change in ONS76 cell line depleted or not for AMBRA1, treated for 48 hrs with the indicated cytotoxic compounds. **(C)** Cell number fold-change in DAOY (Left) and ONS76 (Right) cell line depleted or not for AMBRA1, treated for 24 hrs with the indicated cytotoxic compound. Unless otherwise stated data are presented as mean value ±SD and n refers to biological independent samples. (A) Data were analyzed using One-way ANOVA. (B) Data were analyzed using unpaired Student t-test.
